# Cardiovascular mortality in people with cancer compared to the general population: A systematic review and meta‐analysis

**DOI:** 10.1002/cam4.70057

**Published:** 2024-08-03

**Authors:** Huah Shin Ng, Rosie Meng, Tania S. Marin, Raechel A. Damarell, Elizabeth Buckley, Joseph B. Selvanayagam, Bogda Koczwara

**Affiliations:** ^1^ Flinders Health and Medical Research Institute, College of Medicine and Public Health Flinders University Adelaide South Australia Australia; ^2^ SA Pharmacy, SA Health Adelaide South Australia Australia; ^3^ Research Centre for Palliative Care, Death and Dying, College of Nursing and Health Sciences Flinders University Adelaide South Australia Australia; ^4^ Department of Cardiovascular Medicine Flinders Medical Centre Adelaide South Australia Australia; ^5^ Department of Medical Oncology Flinders Medical Centre Adelaide South Australia Australia

**Keywords:** cancer, cardiovascular disease, meta‐analysis, standardised mortality ratios, systematic review

## Abstract

**Background:**

Cardiovascular disease (CVD) is the leading cause of non‐cancer death in cancer survivors, but the risk of CVD varies between cancers.

**Objectives:**

To synthesise available evidence on patterns and magnitude of CVD mortality risk.

**Methods:**

A systematic search of Medline (OVID), CINAHL and Scopus databases from 01‐January‐2000 to 16‐July‐2023 of studies of people with cancer, reporting CVD mortality in cancer population compared with a reference population (e.g. general population) as standardised mortality ratios (SMR). Meta‐analysis of SMRs across cancer and CVD types were pooled using a random‐effects model to allow for heterogeneity of the true effect size across studies.

**Results:**

We identified 136 studies from 16 countries. Sample sizes ranged from 157 to 7,529,481. The majority (*n* = 98; 72%) were conducted in the United States, followed by Europe (*n* = 22; 16%). The most common cancers studied were gastrointestinal (*n* = 34 studies), haematological (*n* = 31) and breast (*n* = 29). A total of 876 CVD SMRs were extracted across diverse CVD conditions. Of those, the majority (535; 61%) indicated an increased risk of CVD death (SMR >1), 109 (12%) a lower risk of CVD death (SMR <1) and 232 (27%) an equivalent risk (95% CI of SMR included 1) compared to the general population. The meta‐analysis of all reported SMRs showed an increased risk of CVD death (SMR = 1.55, 95% CI = 1.40–1.72) in cancer survivors compared with the general population. The SMR varied between CVD conditions and ranged from 1.36 (95% CI = 1.29–1.44) for heart diseases to 1.56 (95% CI = 1.39–1.76) for cerebrovascular diseases. SMR varied across cancer types, ranging from 1.14 (95% CI = 1.04–1.25) for testicular/germ cell tumours to 2.82 (95% CI = 2.20–3.63) for brain/central nervous system tumours.

**Conclusions:**

Cancer survivors are at increased risk of premature CVD mortality compared to the general population, but the risk varies by cancer type and CVD. Future research should focus on understanding mechanisms behind the increased CVD risk to develop appropriate interventions.

## Graphical abstract

This study has summarised the existing evidence demonstrating higher cardiovascular (CVD) mortality in people with cancer compared to the general population across different CVD conditions and different cancer types. Reducing CVD mortality after cancer diagnosis should be an important priority for survivorship research and care.

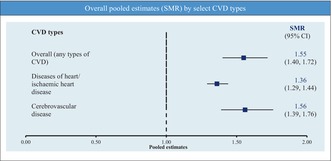

## 1 INTRODUCTION

Cardiovascular disease (CVD) and cancer are the leading causes of mortality and morbidity worldwide,^1^ with growing evidence that cancer survivors are at higher risk of developing CVD and have higher CVD mortality than the general population.^2^, ^3^, ^4^ This increased risk of CVD may be explained by shared risk factors including smoking, obesity, physical inactivity, poor diet intake and excessive alcohol consumption.^5^ It may also be a result of adverse effects of cancer treatment, such as chemotherapy, radiotherapy, immunotherapy or targeted therapy, which have been associated with cardiovascular complications.^6^, ^7^ Further, cancer survivors appear to be less likely to adhere to the prescribed CVD risk factor‐related medications.^8^

Diseases of circulatory system comprise a range of conditions that affect heart and blood vessels including cardiovascular (diseases of blood vessels and/or the heart) and cerebrovascular diseases (diseases of the blood vessels in the brain).^9^, ^10^, ^11^ These conditions are interconnected due to underlying pathology and are often grouped under the umbrella term of CVDs.^11^ While CVD is recognised as a major cause of non‐cancer death among long‐term cancer survivors,^4^, ^12^ there is variability in the published data regarding magnitude of risk for CVD death, and no meta‐analysis available to date that has synthesised and quantified the risk of CVD mortality in different populations of cancer survivors and according to different types of CVD. Therefore, the aim of this study was to conduct a systematic review and meta‐analysis of available research to compare the pattern and magnitude of CVD mortality between individuals with cancer and the general population.

## 2 METHODOLOGY

We conducted a systematic review and meta‐analysis according to the Preferred Reporting Items for Systematic Reviews and Meta‐Analyses (PRISMA) checklist^13^ to address the question: What is the risk of non‐cancer death from CVD in people with cancer compared with the general population, and does the risk differ by cancer types?

### 2.1 Search strategy and selection criteria

Studies were eligible if they included people of all ages with a diagnosis of cancer (any type) and reported CVD mortality‐related data in the form of standardised mortality ratio (SMR). Papers were eligible if peer‐reviewed, in English and published since 2000. Eligibility also allowed a broad range of study designs to be considered including cohort studies (prospective or retrospective), case–control studies, and case series. Unpublished manuscripts, conference abstracts and posters, letters, commentaries and editorials were excluded. Studies with a focus on paediatric populations (defined as cancer diagnosed among children aged 0–14 years) were excluded as the types of cancers reported in children and their needs often differ from those observed in adults.^14^, ^15^ Studies that included people with second cancer at adult age who had been treated for paediatric cancers years ago were not an exclusion criterion for our review.

We searched three databases (Medline (OVID), CINAHL (EBSCOhost) and SCOPUS) from 1 January 2000 to 16 July 2023 using a highly sensitive strategy comprising a range of search terms for each of the three key concepts (1) cancer, (2) mortality and (3) SMR (see Supplementary File—File S1 for search strategy). We also scanned the reference lists of all included studies for additional studies not identified by the database search.

Title and abstract screening was conducted independently by two authors, followed by full‐text screening for eligibility to be included in the data synthesis. Any disagreements were resolved by consensus with the third author.

We registered the study protocol with the Prospective Register of Systematic Reviews database (Registration number: PROSPERO 2020 CRD42020209215).

### 2.2 Data analysis

The outcome of interest was the risk of non‐cancer death from CVD expressed as an SMR reported by primary study authors. The SMR measures the ratio of the number of deaths from CVD observed in the cancer population over a given period to the number that would be expected in the wider reference population (e.g. general population with similar distribution of age, and/or sex or controls from the same study setting without a cancer) over the same period.^16^ If an SMR is greater than one, it is interpreted as excess of mortality from CVD in the cancer population. Non‐cancer death from CVDs were identified using International Classification Diseases code with diagnoses captured in hospital data, cancer registry or death records as reported by primary study authors, although the definitions were not clearly specified in some studies. All terms related to CVDs were considered in our study and grouped into nine broad categories: (i) diseases of the circulatory system (including all CVDs such as cardiovascular and cerebrovascular diseases, type specified/unspecified as reported by study authors); (ii) diseases of heart/heart disease (comprising a range of conditions in combination such as rheumatic heart disease, hypertensive heart disease, ischemic heart disease, pulmonary heart disease, other forms of heart disease); (iii) ischaemic heart disease (including other terms such as acute myocardial infarction, coronary heart disease, other ischaemic diseases of the heart); (iv) heart failure (including other terms such as combination of cardiomyopathy/congestive heart failure as reported by primary study authors); (v) hypertension (including other terms such as hypertensive diseases, hypertension without heart disease); (vi) atherosclerosis; (vii) aortic aneurysm and dissection; (viii) cerebrovascular disease/stroke (including other terms such as ischaemic/haemorrhagic stroke); and (ix) others (e.g. arrythmia, chronic rheumatic heart disease, embolism/thrombosis, valvular heart disease/heart valve disease, noncardiac circulatory diseases, other CVDs).

Data were extracted from the eligible studies by two authors using a standardised extraction form and included: first author surname, publication year, study country, study design, data source, study period, number of study population, demographics (sex, age and ethnicity/race), follow‐up duration, cancer types and SMR and its corresponding 95% confidence intervals (CIs) by type of CVDs.

We presented the SMRs by type of CVDs and by 15 broad categories of cancer sites (all cancers [e.g. multiple cancers combined as reported by the study author], breast, gastrointestinal [e.g. colorectal, gastric, pancreatic, liver, anus], prostate, lung, haematological [e.g. lymphoma, leukaemias], gynaecological [e.g. ovarian, endometrial, cervix], testicular and other germ cell tumours, other urological [e.g. bladder, kidney], head and neck [e.g. oral, salivary gland], sarcomas, thyroid cancers, brain and other central nervous system (CNS) tumours, skin and melanoma and other cancers [e.g., neuroblastoma, retinoblastoma, neuroendocrine tumours]).

The quality of each study was assessed independently by two authors using the Newcastle‐Ottawa Scale (NOS) for evaluating non‐randomised studies in systematic reviews and meta‐analyses.^17^ Any disagreements were resolved by consensus with the third author who did not have a direct involvement in data extraction and the computation of meta‐analysis. The risk of bias assessment included: selection bias (representativeness of the cancer cohort, selection of the general population (non‐cancer cohort), ascertainment of cancer status, demonstration that CVD death was not present at the start of the study), comparability (study controls for sex and age on the basis of the design or analysis), and outcome (assessment of outcome, duration of follow‐up for outcome to occur, and adequacy of follow‐up of cohorts). A total of nine scores can be given for each study. No study was excluded from the review based on quality and risk of bias assessment scores that ranged from 6 to 9 (Supplementary Material—File S4).

We conducted the meta‐analysis using Stata 17 statistical software (17.0. StataCorp LLC, College Station, TX). All reported SMRs across all cancer and CVD types (any of the nine broad CVD categories) were pooled using random‐effects models to allow for heterogeneity of the true effect size across studies. We also reported the overall pooled estimate and 95% CIs by broad categories of cancer sites for two select types of CVDs: diseases of heart/heart disease/ischemic heart disease and cerebrovascular disease/stroke. The summary effect sizes were calculated using a DerSimonian‐Laird random‐effect model in Stata.

## 3 RESULTS

**FIGURE 1:**
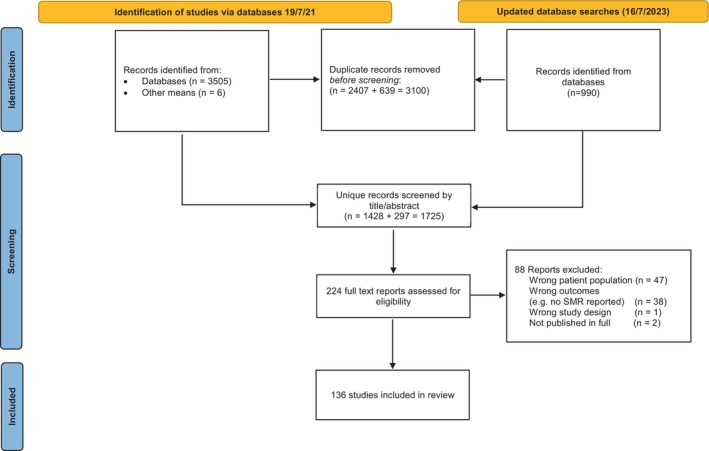
Flow diagram of study selection. A total of 136 studies were included in the review.

Database search was first run on 19 July 2021 and then updated on16 July 2023. We retrieved 4501 records across both sets of search, which reduced to 1725 records once duplicates were removed. After full‐text screening, a total of 136 studies met the inclusion criteria (Figure 1). Studies included in the systematic review covered 16 countries spanning four continents. Most (*n* = 98; 72%) were conducted in the United States, followed by 22 studies in Europe (16%), four studies each in Australia and in South Korea, three studies in Japan, one study each in China and Canada, and three cross‐national (Table S1). None of the studies were conducted in Africa or South America. Study publication years ranged from 2000 to 2023, with over three‐quarters (*n* = 105) published in the last 5 years (2019–2023). All but two studies (*n* = 134; 99%) were retrospective cohort studies (one was a prospective cohort study, and one used secondary data from participants enrolled in a randomised screening trial). The most common data sources used were the Surveillance, Epidemiology and End Results (SEER) data (*n* = 95; 70%), followed by the use (at least in part) of a cancer registry (*n* = 29; 21%). The study period varied across studies ranging from years 1943–2002 to 2005–2020.

The study sample sizes ranged from 157 to 7,529,481 people across all ages with half of the study populations being female (Table S1). Five studies restricted the study population to adolescents or young adults (ages ranged from 15 to 39 years). The most common broad categories of cancer sites studied were gastrointestinal cancer (reported at least in part in 34 studies), followed by haematological cancer (reported at least in part in 31 studies), breast (*n* = 29), urological (*n* = 25), gynaecological (*n* = 23) and prostate cancer (*n* = 22).

A total of 876 SMRs were reported for nine broad categories of CVDs across 15 cancer sites (Table S2). Of those, 535 (61%) indicated an increased risk of CVD death (SMR >1), 109 (12%) a lower risk of CVD death (SMR <1) and 232 (27%) were not significant (the 95% CIs of the SMR included 1 indicating equivalence risk to general population).

The meta‐analysis of all reported SMRs across all cancer and CVD types showed an increased risk of CVD death (SMR = 1.55, 95% CI = 1.40–1.72) in the cancer population compared with the general population (Table 1). The results remained significant across two broad types of CVDs including diseases of heart/heart disease/ischaemic heart disease (SMR = 1.36, 95% CI = 1.29–1.44) and cerebrovascular disease/stroke (SMR = 1.56, 95% CI = 1.39–1.76).

**TABLE 1:** Overall pooled estimates (SMRs) in people with cancer compared to the general population across all cancer and CVD types, and by select CVD types.

	Number of study points	SMR	95% CI	*p*‐value of effect size	*p*‐value of Cochran's *Q* statistics for heterogeneity	Tau‐squared	% Weight
Overall (any types of CVDs)	876	1.55	1.40–1.72	<0.001	<0.001	2.50	100
By CVD types							
Diseases of heart/ischaemic heart disease	209	1.36	1.29–1.44	<0.001	<0.001	0.16	100
Cerebrovascular disease	155	1.56	1.39–1.76	<0.001	<0.001	0.57	100

Abbreviations: CI, confidence interval; CVD, cardiovascular disease; SMR, standardised mortality ratio.

**FIGURE 2:**
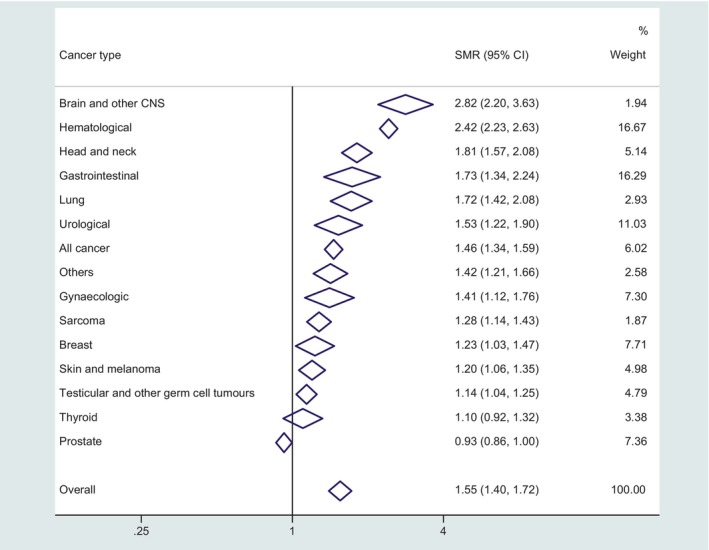
Forest plot showing SMRs (any types of cardiovascular diseases) in people with cancer compared to the general population by cancer type on log scale. The figure shows the SMR and 95% CI by type of cancer. SMR, standardised mortality ratio; CI, confidence interval; CNS, central nervous system.

Compared with the general population, the risk of death from all types of CVD combined was significantly higher across nearly all individual cancer types ranging from 1.14 (95% CI = 1.04–1.25) in testicular and other germ cell tumours to 2.82 (95% CI = 2.20–3.63) in brain and other CNS tumours (Figure 2). The results were not significant for two cancer types including thyroid (SMR = 1.10, 95% CI = 0.92–1.32) and prostate cancer (SMR = 0.93; 95% CI = 0.86–1.00).

Further examination of the SMRs within two broad types of CVDs showed the risks differed by types of cancer (Figures S1 and S2). The risk of mortality from: (i) diseases of heart/heart disease/ischaemic heart diseases ranged from 1.32 (95% CI = 1.11–1.56) for all cancers to 2.46 (95% CI = 2.19–2.77) for haematological cancer (Figure S1); and (ii) cerebrovascular disease/stroke ranged from 1.25 (95% CI = 1.12–1.39) for urological cancer to 5.95 (95% CI = 3.93–9.00) for brain and other CNS tumours (Figure S2).

In term of comparability between cancer and the reference population, the majority of studies (*n* = 117; 86%) comprised reference population controlled for sex and age on the basis of the design or analysis, 15 studies (11%) accounted for either age or sex only, and 4 (*n* = 3%) without the relevant information provided in their published studies (Supplementary Material—File S1).

## 4 DISCUSSION

To our knowledge, this systematic review and meta‐analysis provides the most comprehensive data synthesis of the burden of CVD mortality in cancer populations compared with the general population, comprising data from 136 studies across 16 countries published over last 23 years.

The meta‐analysis showed an overall 55% increase in CVD mortality compared to the general population, with significant variability across types of cancer and CVD. The highest risk relative to the generation population was for brain and other CNS tumours (SMR: 2.82, 95% CI = 2.20–3.63), likely reflecting well recognised higher risk of cerebrovascular disease, but also significantly higher risk of thrombosis in these individuals.^18^, ^19^, ^20^, ^21^, ^22^, ^23^ This finding is of major concern in this population given the high cancer specific mortality creating a ‘double jeopardy’. The risk of mortality from diseases of heart/ischemic heart diseases was highest for haematological cancer, likely relating to cancer drug and radiation‐induced cardiotoxicity.^24^

In two cancer types—thyroid and prostate—there was no significant difference in risks of CVD mortality compared with the general population. For thyroid cancer, this finding may be explained by the lack of cardiotoxic cancer treatment as most thyroid cancers are treated with surgery.^25^ It is also possible that the risk of CVD death may be influenced by differences in treatment and tumour stage. With regard to prostate cancer, the same reason may apply in case of early‐stage cancers which may be treated with surgery, radiation or watchful waiting only but may not be applicable to distant/metastatic cancers treated with known cardiotoxic therapies such as androgen deprivation therapy.^26^, ^27^, ^28^, ^29^ The use of androgen deprivation therapy is known to be associated with adverse cardiovascular events, but the recent meta‐analysis showed that the effects were not consistently reproducible.^30^ The lack of increased CVD risk in individuals with advanced cancer may reflect older age of prostate cancer survivors where the baseline general population risk of CVD is also higher and subjectivity of death records where potential CVD death in an individual with a history of prostate cancer may be attributed to prostate cancer rather than CVD.

There was significant variability of risk according to cancer type, which may reflect differences in the distribution of cardiotoxic treatments^31^, ^32^, ^33^, ^34^ and also background CVD risk factors across cancers.^5^ Several anticancer medicines including anthracyclines, alkylating agents (e.g. cyclophosphamide), taxane (e.g. paclitaxel), monoclonal antibodies (e.g. trastuzumab), and tyrosine kinase inhibitors (e.g. sunitinib) are linked to adverse cardiovascular events such as cardiac dysfunction and heart failure.^31^, ^32^, ^33^, ^34^ For example, the higher risk of all CVD deaths among people with haematological cancers (SMR: 2.42; 95% CI = 2.23–2.63) could be linked to the cardiotoxic effects of cancer therapy used to treat blood cancers such as lymphoma and leukaemias.^35^, ^36^, ^37^, ^38^, ^39^, ^40^ It is notable that higher SMRs were observed in cancers that share CVD risk factors^29^, ^41^, ^42^, ^43^ such as head and neck, lung and gastrointestinal cancers whereas cancers that are recognised for high exposure to cardiotoxic treatments, such as breast cancer were associated with lower SMR (but still higher than general population). This finding underscores the importance of assessment and management of pre‐existing CVD risk factors including through lifestyle modifications such as increased physical activity, exercise, weight management, healthy diet and smoking cessation in order to reduce CVD risk.^44^, ^45^, ^46^

When examining by CVD type, the risk of CVD death remained significantly elevated across all cancers, ranging from a 36% higher risk for diseases of heart/heart disease/ischemic heart disease, to 56% for cerebrovascular disease/stroke. The risk of death from cerebrovascular diseases was highest for people with brain/other CNS tumours (SMR: 5.95; 95% CI = 3.93–9.00) followed by gastrointestinal (SMR: 2.31; 95% CI = 1.35–3.95) and head and neck cancers (SMR: 1.81, 95% CI = 1.17–2.86) than the general population, which may be due to the direct effects of tumours on cerebral vasculature or cancer‐associated hypercoagulable state caused by tumours,^18^, ^19^, ^20^, ^21^, ^22^, ^47^ or the effects of cardiotoxic cancer treatment that can affect cerebral vasculature either through drug effects or radiation over carotid vessels as well as shared risk factors.^18^, ^22^, ^48^, ^49^ Given that the growing field of cardio‐oncology is focused predominately on conditions of the heart, the findings of high risk of cerebrovascular disease death warrant further attention in terms of future research and practice. Historically, the field of cardio‐oncology was well advanced in the management of cancers with good prognosis such as breast cancer and lymphoma. Our data suggests that there is a need for cardio‐oncology focus in other tumours such as brain/CNS tumours who might have a much shorter life expectancy on average, but there may still be benefits for early CVD preventive interventions given high risk of premature CVD mortality.^18^ It is possible that in this population with worse cancer prognosis, management of CVD risk factors is not well‐addressed amplifying the risk for CVD. An interdisciplinary team approach for risk reduction and early prevention may help reduce CVD mortality.^50^, ^51^ The variability of SMRs between CVD and cancer types may also reflect how these conditions were reported. Within this study we categorised CVD into nine broad categories with some overlap of conditions—for example it is likely that there is significant overlap between categories of any CVD and diseases of the circulatory system. Future research and routine data monitoring should adopt standardised categories of CVD conditions and systems of reporting (e.g. there should be clearer reporting international classification of diseases codes in primary research using either administrative or clinical records) to ensure greater precision of future data analyses.

This study has several limitations. The interpretation of CVD data was challenging at times, particularly when the individual types of CVDs included in the original articles were not clearly defined. Therefore, we included all types of CVDs reported by study authors to provide an overview of the burden of CVD in cancer populations. Most of the studies included in the review were from the United States, and hence, the generalisability of the current literature to other countries across diverse racial and ethnic groups remains to be explored. It is possible that the risks of CVD may vary across countries due to differences in standard approaches and guidelines on the management of cancer, and differences in the availability of medical treatment for cancer detection and management of accompanying complications.^18^ Although we pooled all SMRs to provide an overall risk of CVD death, there were differences in study design, study population (e.g. different cancer stages and comorbidity burden, exposure to different cancer treatment such as chemotherapy, immunotherapy, radiotherapy), sample sizes and study period (e.g. different calendar years or duration of follow‐up) across studies that may warrant further subgroup analysis. The variability in data reflects heterogeneity of populations including types of cancer treatments, time from treatment and adherence to treatment, comorbidities, pre‐existing risk factors and other relevant clinical information, which were often not available for analysis. We also cannot rule out the potential misclassification of causes of death based on mortality statistics alone (versus autopsy as the gold standard for the correct diagnosis).^52^, ^53^ Owing to the nature of the data (i.e. all but two studies included in the review were retrospective cohort studies), we were not able to infer causality for the identified associations.

Irrespective of these limitations, this study has summarised the vast evidence and demonstrated higher mortality from CVD in people with cancer compared to the general population. This increased mortality is observed across different CVD conditions and cancer types making it an important priority for survivorship research and care. There is a need for greater understanding of the mechanisms of increased risk, which seem to extend beyond just a direct toxic effect of cancer therapy, as this greater understanding of mechanisms (e.g. genetic predisposition, and molecular, biological and environmental factors through a range of pre‐clinical, clinical and translational research) can direct development of appropriate interventions.^44^

Fundamental to the development of interventions is the need for better assessment of risk of CVD mortality and this is dependent on accurate data reporting, not just in research but in routine data monitoring of cancer burden. There is a need for the development of standardised data reporting systems and datasets to enhance the comparability of data. With the advancement in big data analyses including machine learning, future analyses could incorporate complex information including treatment history and comorbidity data to further refine data analyses.

In conclusion, cancer survivors are at increased risk of premature CVD mortality compared to the general population, but the risk varies by type of cancer and CVD. Future research should focus on understanding mechanisms behind increased risk to develop appropriate targeted interventions.

## AUTHOR CONTRIBUTIONS

**Huah Shin Ng:** Conceptualization (lead); data curation (lead); formal analysis (supporting); investigation (lead); methodology (lead); validation (lead); visualization (supporting); writing – original draft (lead); writing – review and editing (lead). **Rosie Meng:** Conceptualization (supporting); formal analysis (lead); methodology (supporting); visualization (lead); writing – review and editing (supporting). **Tania S. Marin:** Conceptualization (supporting); data curation (supporting); investigation (supporting); methodology (supporting); validation (supporting); writing – review and editing (supporting). **Raechel A. Damarell:** Conceptualization (supporting); data curation (supporting); methodology (supporting); visualization (supporting); writing – review and editing (supporting). **Elizabeth Buckley:** Conceptualization (supporting); methodology (supporting); writing – review and editing (supporting). **Joseph B. Selvanayagam:** Conceptualization (supporting); methodology (supporting); writing – review and editing (supporting). **Bogda Koczwara:** Conceptualization (lead); methodology (lead); supervision (lead); writing – original draft (lead); writing – review and editing (lead).

## FUNDING INFORMATION

There was no funding for this study.

## CONFLICT OF INTEREST STATEMENT

The authors have no relevant financial or non‐financial interests to disclose related to the conduct of this study.

## Supporting information


Figure S1.

Table S1.

Table S2.

Table S3.

File S1.

## Data Availability

All data used for the study has been included in the manuscript and supplementary material.
