# Supplementary material for: Cardiovascular mortality in people with cancer compared to the general population: A systematic review and meta‐analysis
Source: Cancer Med. 2024 Aug 3;13(15):e70057. doi: 10.1002/cam4.70057 (PMC11297437; doi:10.1002/cam4.70057)
Supplement: Supplementary file 1 — Figure S1. [file CAM4-13-e70057-s005.docx]

**Supplementary Figure 1** Forest plot showing SMRs for diseases of heart/heart disease/ischemic heart disease in people with cancer compared to the general population by cancer type on log scale

**Supplementary Figure 2** Forest plot showing SMRs for cerebrovascular disease in people with cancer compared to the general population by cancer type on log scale
