# Supplementary material for: Cardiovascular mortality in people with cancer compared to the general population: A systematic review and meta‐analysis
Source: Cancer Med. 2024 Aug 3;13(15):e70057. doi: 10.1002/cam4.70057 (PMC11297437; doi:10.1002/cam4.70057)
Supplement: Supplementary file 2 — Table S1. [file CAM4-13-e70057-s004.docx]

**Supplementary Table 1.** Summary of the included studies and patient characteristics

| **First author, year of publication, location** | **Data source** | **Study time period** | **Study population size (number of women; %)** | **Age range at diagnosis/ mean/ median (years)** | **Race/ ethnicity** | **Follow-up duration** | **Cancer (broad category)** | **Cancer type, stage (if specified) or treatment-specific (if specified)** |
| --- | --- | --- | --- | --- | --- | --- | --- | --- |
| **North America (1 Canada, 98 United States)** | | | | | | | | |
| Arora 2019,^1^ British Columbia, Canada | Cancer Registry British Columbia, vital statistics data | 1990-2014 | 6,427  (6,427; 100%) | All ages;  Mean: 63  Median: 60 | NR | Range: 0 to >10 years | Gynaecological | Epithelial ovarian |
| Abuamsha 2019,^2^ United States | SEER | 2000-2015 | 153,983 (69,059; 45%) | ≥20;  Mean: 66.24 | White: 87%  Other: 13% | 37 months (median) | Haematological | Non-Hodgkin lymphoma:  Chronic lymphocytic leukemia/small lymphocytic lymphoma,  Diffuse large B‐cell lymphoma,  Follicular lymphoma |
| Afifi 2020,^3^ United States | SEER | 2000-2015 | 754,270 (754,270; 100%) | All ages | White: 81%  Black: 11%  Asian: 8%  American Indian/ Alaska Native: 0.4% | Range: <1 to >10 years | Breast | Breast |
| Afifi 2021,^4^ United States | SEER | 2000-2015 | 302,345 (145,514; 48%) | ≥20 | White: 80%  Black: 11%  Asian: 8%  American Indian/ Alaska Native: 0.5% | Range: 0 to > 10 years | Gastrointestinal | Colorectal, Stage I-III |
| Afify 2023,^5^ United States | SEER | 2000-2018 | 44,028 (8,508; 19%) | All ages | White: 81%  Black: 16%  Asian: 3%  American Indian/ Alaska Native:  0.4% | Range: 0 to >10 years | Head and neck | Laryngeal |
| Agha 2022,^6^ United States | SEER | 1992-2014 | 1,059,048 (1,059,048; 100%) | All ages | White: 82%  Black: 10%  Other:  7%  Unknown: 0.6% | Range: 0 to >10 years | Breast | Breast, treatment-specific (chemotherapy vs. radiotherapy vs. chemotherapy and radiotherapy) |
| Anderson 2019,^7^ United States | SEER | 1973-2015 | 235,641 (142,551; 60%) | 15-39 | White: 82%  Black: 10%  Other: 8% | Total: 3.1 million total person-years | All cancer  (incl. Breast, Gynaecological, Brain and other CNS, Gastrointestinal, Head and neck, Haematological, Skin, Sarcoma, Testicular and other germ cell tumours, Thyroid) | All cancer, >2 month-survivors:  Breast,  Cervix/ uterus,  CNS tumours,  Colorectal,  Head and neck,  Hodgkin lymphoma,  Leukemias,  Melanoma,  Non-Hodgkin lymphoma,  Soft tissue sarcoma,  Testicular,  Thyroid |
| Anderson 2021,^8^ United States | SEER | 2000-2017 | 183,153 (183,153; 100%) | ≥15 | White: 81%  Black: 10%  Other:  9% | Range: <1 year to >10 years | Gynaecological | Endometrial |
| Appiah 2021,^9^ United States | SEER | 2000-2016 | 1,139,767 (1,139,767; 100%) | ≥20;  Mean: 60.3 | White: 70%  Black: 10%  Hispanic: 11%  Other: 9% | 5.1 years (median) | All breast and gynaecological cancers | Breast,  Cervix,  Gynaecological cancers,  Other female reproductive organs,  Ovary,  Uterus |
| Awad 2022,^10^ United States | SEER | 2000-2018 | 235 (116; 49%) | Mean: 49.2 | White: 75% | 0-5 years | Others | Cardiac |
| Beard 2013,^11^ United States | SEER | 1973-2006 | 9,193  (0; 0%) | 15-70 | White: 94%  Non-white:  6% | 12.3 years (median) | Testicular and other germ cell tumours | Testicular seminoma, Stage I |
| Chen 2020,^12^ United States | SEER | 1975-2016 | 834,510 (406,704; 49%) | All ages;  Mean: 67.2 | White: 81%  Black: 11%  Other: 8% | 3.7 years (median)  Range: 0-41.9 years | Gastrointestinal | Colorectal |
| Chen 2021,^13^ United States | SEER | 2004-2016 | 82,454  (40,170; 49%) | ≥40 | White: 77%  Black: 13% Other: 10% | NR | Lung | Lung |
| Chen 2022a,^14^  United States | SEER | 2004-2015 | 5,925 (4,212; 71%) | ≥18 | White: 76%  Black: 12%  Other: 12% | NR | Gastrointestinal | Gallbladder |
| Chen 2022b,^15^  United States | SEER | 1975-2016 | 80,905 (36,283; 45%) | All ages | White: 81%  Black: 11%  Asian: 7%  American Indian/ Alaska Native:  0.7%  Unknown: 1% | Range: 0 to >10 years | Sarcoma | Bone/ soft tissue |
| Chen 2022c,^16^  United States | SEER | 1975-2016 | 683,333 (305,627; 45%) | All ages | White: 83%  Black: 10%  Other: 7% | 3 years (median)  Range: 0-42 years | Haematological | Haematological (including acute lymphoid leukemia, acute myeloid leukemia, chronic lymphocytic leukemia, chronic myeloid leukemia, Hodgkin lymphoma, plasma cell myeloma, non-Hodgkin lymphoma) |
| Chen 2023,^17^ United States | SEER | 1975-2018 | 36,013 (16,762; 47%) | 20-49 | White: 75%  Black: 14%  Other: 11% | 15.2 years (mean) | Gastrointestinal | Colorectal, early-onset |
| Cheng 2022,^18^ United States | SEER | 2000-2015 | 153,642 (153,642; 100%) | All ages | White: 82%  Black: 10%  Asian: 8%  American Indian/ Alaska Native: 0.6% | Range: <1 to >10 years | Gynaecological | Endometrial |
| Chi 2023,^19^ United States | SEER | 2007-2016 | 2,497 (1,168; 47%) | All ages | White: 78%  Black: 9%  Other: 13% | 4.5 years (mean) | Haematological | Primary CNS lymphoma/ Primary CNS lymphoma-diffuse large b-cell lymphoma |
| Dai 2022,^20^  United States | SEER | 1973-2015 | 563,298 (276,592; 49%) | All ages; Median: 68.6 | White: 83%  Black: 10%  Other: 7% | 6.8 years (median) | Gastrointestinal | Colorectal |
| Dalal 2020,^21^ United States | SEER | 2000-2016 | 7,289  (3,023; 41%) | All ages;  Mean: 69.7 | White: 88%  Black: 5%  Other: 5%  Unknown: 2% | 5.2 years (mean) | Haematological | Lymphoplasmacytic lymphoma/Waldenstrom macroglobulinemia |
| Dores 2020,^22^ United States | SEER | 2000-2016 | 20,007  (9,355; 47%) | 20-74 | NR | 8.0 years (mean) | Haematological | Hodgkin lymphoma, treatment-specific (initial chemotherapy) |
| Du 2021,^23^ United States | SEER | 1975-2016 | 173,710  (133,276; 77%) | ≥15;  Median: 48 | White: 81%  Black: 6%  Other: 11%  Unknown: 1% | 101 months (median) | Thyroid | Papillary Thyroid, Follicular Thyroid,  Hurthle cell Thyroid,  Medullary Thyroid,  Anaplastic Thyroid |
| Eifler 2012,^24^ United States | Single institution data and national death index | 1975-2009 | 18,209 (0; 0%) | Median: 59 | White: 88%  Black: 8%  Other: 4% | 7.4 years (median) | Prostate | Prostate, treatment-specific (radical prostatectomy) |
| Elgenidy 2022,^25^ United States | SEER | 2000-2018 | 8,962 (4,533; 51%) | All ages | White: 78%  Black: 8%  Asian: 13%  American Indian/ Alaska Native: 0.8% | Range: <1 to >10 years | Gastrointestinal | Intrahepatic cholangiocarcinoma |
| Elgenidy 2023,^26^ United States | SEER | 2000-2018 | 165,969 (61,012; 37%) | All ages | White: 82%  Black: 11%  Asian: 5%  American Indian/ Alaska Native: 0.9% | Range: 0 to >10 years | Urological | Renal |
| Elmehrath 2021a,^27^ United States | SEER | 2000-2016 | 26,168 (0; 0%) | All ages  Mean: 71 | White: 74%  Black: 19%  Asian: 6%  American Indian/ Alaska Native: 0.6% | Range: <2 years to > 5 years | Prostate | Prostate, metastatic |
| Elmehrath 2021b,^28^ United States | SEER | 2000-2016 | 82,590  (82,590; 100%) | All ages | White: 83%  Black:  8%  Asian:  8%  American Indian/ Alaska Native: 0.6% | Range: <1 year to >10 years | Gynaecological | Ovarian |
| Fang 2010,^29^ United States | SEER | 1979-2005 | 342,497  (0; 0%) | All ages;  Mean: 70.2 | White: 83%  Black: 11%  Others: 5%  Unknown: 1% | Total: 12 months | Prostate | Prostate, first-year after diagnosis |
| Felix 2017,^30^ United States | SEER | 1988-2012 | 157,496  (157,496; 100%) | ≥15;  Mean: 62 | White: 84%  Black: 8%  Other: 7%  Unknown: 0.5% | Range: <5 years to >10 years | Gynaecological | Endometrial |
| Feng 2021,^31^ United States | SEER | 2000-2016 | 475,771 (230,199; 48%) | All ages | White: 79%  Black: 12%  Other: 9% | Range: <1 year to >10 years | Gastrointestinal | Colorectal |
| Fung 2015,^32^ United States | SEER | 1980-2010 | 15,006 (0; 0%) | All ages | White:  94%  Non-white: 6% | Surgery:  7.9 / 10 years (median/ mean) Chemotherapy:  6.5 / 8.7 years (median/ mean) | Testicular and other germ cell tumours | Testicular nonseminoma, treatment-specific (surgery only vs. chemotherapy only as initial treatment) |
| Gad 2019,^33^ United States | SEER | 2000-2014 | 4,671,989 (NR) | All ages;  Mean: 75.5 | White: 73%  Black:  11%  Asian:  6%  American Indian/ Alaska Native:  0.4%  Hispanic:  10% | Range: <1 year to 10 years | All cancer (incl. Breast, Gastrointestinal, Urological, Haematological, Lung, Skin, Head and neck, Prostate, Thyroid, Gynaecological) | All cancer:  Breast, Colorectal,  Kidney and renal pelvis,  Leukemias, Liver, Lung, Melanoma, Non-Hodgkin lymphoma, Oral cavity and pharynx, Pancreatic  Prostate, Thyroid, Urinary bladder, Uterine |
| Gaitanidis 2019,^34^ United States | SEER | 2010-2014 | 164,719  (77,870; 47%) | All ages;  Mean: 67 | White: 78%  Black: 12%  Other: 9% | 18 months (median) | Gastrointestinal | Colorectal |
| Guan 2021,^35^ United States | SEER | 2004-2015 | 131,306  (131,306; 100%) | ≥45 | White: 81%  Non-white: 18%  Unknown: 1% | 63.8 months (mean);  Range: 63.6-64.1 months | Breast | Breast, treatment-specific (without chemotherapy or RT) |
| Guan 2022,^36^ United States | SEER | 1975-2018 | 80,042 (22,408; 28%) | ≥65 | White: 91%  Black: 4%  Other: 5% | 17.2 years (mean); ≥2months | Urological | Bladder |
| Guan 2023,^37^ United States | SEER | 1975-2018 | 1,141,675 (513,026; 45%) | ≥65 | White: 85%  Black: 8%  Other: 7% | 13.5 years (median);  ≥2months | All cancer | 16 cancer sites |
| Guo 2020,^38^ United States | SEER | 2000-2015 | 389,962  (0; 0%) | ≥15  Mean: 67.55/ 60.52 | White:  80%  Black:  14%  Other:  5%  Unknown: 1% | 93–98 months (median) | Prostate | Prostate, treatment-specific (prostatectomy vs. radiotherapy) |
| Guo 2022,^39^ United States | SEER | 2000-2016 | 752,352 (0; 0%) | All ages | White: 77%  Black: 15%  Other: 5%  Unknown: 3% | Range: <1 to >10 years | Prostate | Prostate |
| Harvitkar 2021,^40^ United States | SEER | 1975-2016 | 67,694 (32,809; 48%) | All ages  (93% aged ≥ 50 years) | White:  80%  Black:  13%  Other:  7% | Range:  <1 to >10 years | Gastrointestinal | Pancreatic |
| Hisada 2007,^41^ United States | SEER | 1973-2002 | 3,104  (712, 23%) | All ages;  Mean: 57.4 | White:  89%  Black:  3%  Other/unkwon:  4%  Hispanic:  4% | 6.5 years (mean);  Range:  2 months to 29.3 years | Haematological | Hairy cell leukemia, 2-month survivor |
| Howlader 2017,^42^ United States | SEER | 2002-2012 | 18,047  (8,432, 47%) | ≥20;  Median: 66 | White: 80%  Black: 7%  Other: 13% | Range: 0-59 months to ≥ 60 months  (max 11 years) | Haematological | Diffuse large B‐cell lymphoma |
| Lee 2000,^43^ United States | Single hospital data | 1970-1999 | 210  (98; 47%) | All ages | NR | 15.6 years (median)  Range : 0.35–26.5 years | Haematological | Hodgkin lymphoma, Stage I-III, treatment-specific (treated with curative radiotherapy) |
| Li 2019,^44^ United States | SEER | 1973-2015 | 44,292  (19,897; 45%) | All ages;  Mean: 53.56 | White: 82%  Black: 9%  Other: 9%  Unknown: 0.3% | 0-15 years | Haematological | AML, treatment-specific (treated with chemotherapy) |
| Liu 2022,^45^ United States | SEER | 1975-2014 | 2,214,944  (1,109,917; 50%) | All ages | White:  83%  Black:  9%  Other:  7%  Unknown: 0.6% | Range: 2 months to >120 months | All cancer  (incl. Brain and other CNS, Breast, Gynaecological, Gastrointestinal, Urological, Head and neck, Lung, Skin, Prostate, Sarcoma, Testicular and other germ cell tumours, Thyroid) | 16 cancer sites, >100,000 person-years survivors (>2 month):  Brain, Breast, Cervix uteri, Colorectal, Corpus uteri, Kidney and renal pelvis, Larynx, Lung and bronchus, Melanoma, Ovary, Prostate, Soft tissue including heart, Stomach, Testis, Thyroid  Urinary bladder |
| Liu 2023,^46^ United States | SEER | 2004-2015 | 576,713 (249,865; 43%) | All ages | White: 78%  Black: 11%  Asian: 10%  American Indian/ Alaska Native:  0.8%  Unknown: 0.4% | 24 months (median) | Gastrointestinal | Colorectal, Gastric, Hepatocellular, Oesophagus, Pancreatic, |
| Lou 2023,^47^ United States | SEER | 1975-2019 | 42,813 (15,725; 37%) | All ages  Mean: 67.7 | White: 68%  Black: 11%  Other: 21% | Range: <1 to >5 years | Gastrointestinal | Gastric |
| Low 2019,^48^ United States | SEER | 1973-2015 | 29,981  (16,037; 53%) | All ages;  Median: 59 | White:  79%  Black:  13%  Other:  8% | Range: <1 to >10 years | Others | Neuroendocrine tumours, >=6 months survivors |
| Lu 2021,^49^ United States | SEER | 2000-2016 | 218,597 who died (NR) | All ages | NR | Range: ≥2 to ≥180 months | Gastrointestinal | Colorectal |
| Lu 2022,^50^ United States | SEER | 1983-2015 | 15,899 (7,150; 45%) | All ages | White: 84%  Black: 10%  Other: 5%  Unknown: 0.6% | Range:  ≥2 to ≥180 months | Haematological | Hodgkin lymphoma |
| Lyu 2022,^51^ United States | SEER | 2000-2017 | 24,040 (24,040; 100%) | ≥15 | White: 88%  Black: 9%  Asian: 3%  American Indian/ Alaska Native:  0.2% | Range:  >2 to >120 months | Urological | Bladder, treatment-specific (treated with local tumour excision or radical cystectomy) |
| Massa 2017,^52^ United States | SEER | 2004-2011 | 64,598  (15,230; 24%) | ≥20;  Mean: 62 | White: 83%  Black: 11%  Other: 5% | 2.1 years (median) | Head and neck | Head and neck |
| Miao 2022,^53^ United States | SEER | 1975-2018 | 39,510 (16,480; 42%) | ≥65 | White: 96%  Black: 0.6%  Other: 1%  Unknown: 2% | 4.17 years (median) | Skin | Melanoma |
| Moke 2021,^54^ United States | SEER | 1975-2016 | 162,317 (101,232; 62%) | 15-39 | White: 83%  Black: 8%  Other:  7%  Unknown: 1% | 17.4 years (median)Range: 5-41.9 years | All cancer (incl. Breast, Sarcoma, Gynaecological, Haematological, Skin, Others, Testicular and other germ cell tumours, Thyroid) | All cancers excl. Kaposi sarcoma, 5-year survivor:  Breast, Bone/ soft tissue sarcoma, Cervical/ uterine, Hodgkin lymphoma, Melanoma, Non-Hodgkin lymphoma, Other, Testicular germ cell tumour, Thyroid |
| Peng 2022,^55^  United States | SEER | 2000-2015 | 10,179 (4,370; 43%) | All ages | White: 87%  Black: 5%  Other: 8% | 39 months (median) | Urological | Bladder (localised vs. regional vs. distant) |
| Sadeq 2023,^56^ United States | SEER | 2000-2019 | 224,624 (98,086; 44%) | All ages | White: 98%  Black: 0.5%  Asian: 0.7%  American Indian/ Alaska Native:  0.2% | Range: >0 to >10 years | Skin | Cutaneous melanoma (localised vs. regional vs. distant) |
| Shi 2021,^57^ United States | SEER | 2007-2016 | 768,055 (555,849; 72%) | 18-64;  Mean: 52.9 | White: 66%  Black: 13%  Other: 21% | Range: 2 months to >60 months | All cancer | 8 cancers (Bladder, Breast, Cervical, Colorectal, Hodgkin lymphoma, Lung, Non-Hodgkin lymphoma Ovarian) |
| Sonbol 2023,^58^ United States | SEER | 2000-2019 | 6,136,803 (2,999,958; 49%) | All ages | White: 82%  Black: 10% | Range: <1 to >10 years | All cancer (incl. Gastrointestinal, Urological, Brain and other CNS, Breast, Gynaecological, Others, Head and neck, Haematological, Lung, Skin, Prostate, Thyroid) | All cancer (Anus, Bladder, Brain, Breast, Cervix, Colorectal, Corpus and uterus, Endocrine system, Kidney, Larynx, Leukemia, Liver, Lung, Lymphoma, Melanoma, Myeloma, Oral cavity and pharynx, Oesophagus, Ovary, Pancreatic, Prostate, Skin, Stomach, Thyroid) |
| Song 2022a,^59^ United States | SEER | 2004-2015 | 143,008 (34,376; 24%) | ≥15 | White: 90%  Black: 6%  Asian: 4%  American Indian/ Alaska Native:  0.3% | Range: <1 to >10 years | Urological | Bladder (non-muscular invasive vs. non-metastatic muscle invasive vs. metastatic) |
| Song 2022b,^60^ United States | SEER | 2004-2018 | 3,597 (0; 0%) | ≥15 | White: 84%  Black: 10%  Asian:  5%  American Indian/ Alaska Native:  1% | Range: <1 to >10 years | Urological | Penile (localised vs. regional vs. distant) |
| Stoltzfus 2020,^61^ United States | SEER | 1992-2015 | 7,529,481 (3,661,011; 49%) | All ages | White: 82%  Black: 10%  Other: 7%  Unknown: 1% | Range: <1 to >10 years | All cancer (incl. Urological, Breast, Gastrointestinal, Gynaecological, Haematological, Lung, Skin, Head and neck, Prostate, Testicular and other germ cell tumours) | All cancer:  Bladder, Breast, Colorectal, Endometrial, Hodgkin lymphoma, Kidney, Leukemias, Lung, Melanoma, Myeloma, Non-Hodgkin lymphoma, Oral cavity and pharynx, Prostate, Testicular |
| Sturgeon 2019,^62^ United States | SEER | 1973-2015 | 3,234,256  (1,571,392; 49%) | All ages | White: 84%  Black:  9%  Other:  6% | Range: 2 to >240 months | All cancer | 28 cancer sites |
| Su 2022,^63^ United States | SEER | 1975-2016 | 645,818 (297,060; 46%) | All ages | White: 85%  Black: 10%  Asian: 5%  American Indian/ Alaska Native:  0.4% | 2319010 person-years | All cancer (incl. Haematological, Gastrointestinal, Urological, Brain and other CNS, Breast, Gynaecological, Others, Head and neck, Lung, Skin, Prostate, Sarcoma) | Multiple primary cancers:  Acute myeloid leukemia, Anus, Bladder, Brain, Breast, Cervix, Chronic lymphocytic leukemia, Chronic myeloid leukemia, Colorectal, Corpus uteri, Endocrine system, Hodgkin lymphoma, Kidney, Larynx, Liver, Lung, Myeloma, Non-Hodgkin lymphoma, Oesophagus, Oral cavity and pharynx, Ovary, Pancreatic, Prostate, Skin, Small intestine, Soft tissue, Stomach, Testis, Ureter, Vulva) |
| Sun 2021,^64^ United States | SEER | 2000-2015 | 42,027  (21,281; 51%) | ≥18;  Mean: 58.57 | White:  74%  Black: 18%  Other: 8% | 54 months (median)  Range: 0 to >10 years | Others | Gastroenteropancreatic neuroendocrine neoplasms |
| Udkoff 2023,^65^ United States | SEER | 1975-2016 | 9,375 (NR) | 10-84 | NR | 27 months (median) | Skin | Merkel cell carcinoma |
| Vo 2022,^66^ United States | SEER | 1975-2017 | 516,916  (516,916; 100%) | 18-84 | White:  83%  Black:  9%  Other:  8% | 9.59 years (median)  11.65 years (mean) | Breast | Breast, ≥1 year survivors, treatment-specific (received initial surgery, chemotherapy, or radiotherapy) |
| Vo 2023,^67^ United States | SEER | 2000-2018 | 739,557 (739,557; 100%) | 18-84 | White: 71%  Black: 10%  Asian/ Native Hawaian: 8%  Hispanic: 11% | Range:  ≥1 to >10 years | Breast | Breast, ≥1 year survivors |
| Wang 2021a,^68^ United States | SEER | 2010-2016 | 161,966  (NR) | ≥18 | White:  81%  Black:  10%  Other:  10% | 41 months (median) | Breast | Breast, T1-2N0M0 and subtype |
| Wang 2021b,^69^ United States | SEER | 2000-2015 | 68,612  (27,138; 40%) | >14;  Median: 60 | White: 81%  Black: 12%  Other: 6% | Range: 0 year to ≥10 years | Urological | Renal, T1-N0M0 |
| Wang 2022a,^70^ United States | SEER | 1975-2016 | 183,641  (140,744; 77%) | All ages | White: 81%  Black: 7%  Other: 12% | 6.9 years (median)Range: 0-41.9 years | Thyroid | Thyroid |
| Wang 2022b,^71^ United States | SEER | 2000-2016 | 237,563 (57,680; 24%) | All ages  Mean: 70.8 | White: 90% | 49 months (median) | Urological | Bladder |
| Wang 2022c,^72^ United States | SEER | 1975-2016 (1992-2016 for SMR) | 165,433 (64,688; 39%) | All ages | White: 72%  Black: 13%  Other: 14%  Unknown: 0.4% | Range: 0 to >240 months | Gastrointestinal | Gastric |
| Wang 2023,^73^ United States | SEER | 2000-2018 | 240,954 (NR) | All ages | NR | Range: <1 to >5 years | Urological | Bladder |
| Weberpals 2018,^74^ United States | SEER | 2000-2014 | 501,547  (501,547; 100%)  (Sub-population for SMR) | ≥15;  Mean: 59.94 | White: 82%  Black: 10%  Asian: 7%  American Indian/ Alaska Native:  0.5%  Other/unknown: 0.4% | >=10 years | Breast | Breast, 10-year survivor, treatment-specific (received either radio/chemotherapy) |
| Weiner 2021,^75^ United States | SEER | 2000-2016 | 752,092  (0; 0%) | All ages | White:  71%  Black:  16%  Asian:  5%  American Indian/ Alaska Native:  0.3%  Hispanic:  8% | Range: <1 to ≥10 years | Prostate | Prostate (local/regional vs. distant) |
| Wu 2022,^76^ United States | SEER | 2004-2015 | 44,506 (21,985; 49%) | ≥18 | White: 86%  Black: 10%  Others: 4% | Range: <1 to >3 years | Lung | Small cell lung cancer |
| Xia 2022,^77^ United States | SEER | 2000-2016 | 43,739 (9,309; 21%) | All ages | White: 84%  Black: 11%  Other: 5% | Range: <1 to >10 years | Gastrointestinal | Esophagus |
| Xie 2022,^78^ United States | SEER | 2005-2016 | 2,688 (0; 0%) | ≥15  Mean: 64.9 | White: 83%  Black: 10%  Other: 5%  Unknown: 1% | NR | Urological | Penile |
| Xue 2023,^79^ United States | SEER | 2000-2015 | 135,831 (135,831; 100%) | All ages | White: 72%  Black: 8%  Asian: 8%  Hispanic: 11%  American Indian/ Alaska Native:  0.6% | Range: <1 to >10 years | Gynaecological | Endometrial |
| Yang 2021a,^80^ United States | SEER | 2000-2015 | 39,500 (0; 0%) | ≥15 | White: 87%  Black:  7%  Other:  5%  Unknown: 0.5% | NR | Urological | Bladder, treatment-specific (surgery, radio/chemotherapy) |
| Yang 2021b,^81^ United States | SEER | 1990-2016 | 7,366,229  (3,717,559; 50%) | All ages | White: 82%  Black: 10%  Asian: 7%  American Indian/ Alaska Native:  0.6% | Total: 1 year | All cancer (incl. Brain and other CNS, Gastrointestinal, Lung) | All cancer:  Brain, Liver, Lung |
| Yang 2023a,^82^ United States | SEER | 2000-2018 | 40,094 (9,155; 23%) | All ages | White: 67%  Black: 14%  Asian: 17%  American Indian/ Alaska Native:  1.2% | Range: <2 to >5 years | Gastrointestinal | Liver |
| Yang 2023b,^83^ United States | SEER | 2000-2017 | 143,036 (54,764; 38%) | ≥15 | White: 82%  Black: 12%  Asian: 5%  American Indian/ Alaska Native:  0.8% | Range: <5 to >10 years | Urological | Kidney (localised vs. regional) |
| Yao 2023,^84^ United States | SEER | 2000-2019 | 12,058 (5,790; 48%) | ≥18 | White: 68%  Black: 18%  Other: 14% | 67 months (mean)  Range: 1-239 months | Gastrointestinal | Gastrointestinal stromal tumour |
| Ye 2022,^85^ United States | SEER | 1975-2016 | 1,170,489 (0; 0%) | ≥40  Mean: 67.7 | White: 79%  Black: 14%  Other: 7% | 6.9 years (median) Range: 0-41.7 years | Prostate | Prostate |
| Yin 2019,^86^ United States | SEER | 2004-2016 | 13,296  (7,792; 59%) | ≥20;  Mean: 66.5/69.0  Median: 67/70 | White:  86%  Black:  8%  Other:  6% | ≥3 months | Lung | Lung, Stage 1A, treatment-specific (lobectomy vs. sublobectomy) |
| Yin 2022a,^87^ United States | SEER | 1975-2016 | 88,328  (41,289; 47%) | ≥40 | White:  74%  Black:  19%  Other:  6%  Unknown: 0.6% | Range: 0 to >120 months | Haematological | Multiple myeloma |
| Yin 2022b,^88^ United States | SEER | 1992-2016 | 29,686 (13,430; 45%) | 15-39 | White: 80%  Black: 12%  Other: 7%  Unknown: 1% | ≥6 months | Haematological | Lymphoma, treatment-specific (radiation vs. no radiation) |
| Yin 2023,^89^ United States | SEER | 2000-2018 | 7,925 (3,900; 49%) | All ages | Non-Hispanic:  82%  Hispanic:  18% | Range: 60 to 216+ months | Haematological | Acute promyelocytic leukemia and non-acute promyelocytic leukemia AM, >5-year survivor |
| Youn 2014,^90^ United States | SEER | 1973-2007 | 28,844  (14,123; 49%) | 15-39 | White: 79%  Black: 13%  Other: 8% | Range: 1 to ≥ 25 years  Total: 113,206 person-years | Sarcoma | Sarcomas (bone and soft tissue) |
| Yu 2012,^91^ United States | SEER | 2000-2007 | 14,238  (NR) | All ages | NR | Total: first 3 years | Head and neck | Squamous cell carcinoma of the oral cavity or oropharynx |
| Yu 2022,^92^ United States | SEER | 2004-2015 | 106,118 (38,865; 37%) | All ages | White: 82%  Black: 12%  Other: 6% | 68 months (median)  Range: <1 to >10 years | Urological | Renal (Stage I/II vs III vs IV) |
| Zagars 2004,^93^ United States | Single cancer centre | 1951-1999 | 453  (0; 0%) | 16-69;  Median: 34 | White: 97%  Black: 3% | 13.3 years (median)14.9 years (mean)  Range: 1-42.8 years | Testicular and other germ cell tumours | Testicular seminoma, Stage I-II, treatment-specific (postorchiectomy radiation therapy without chemotherapy) |
| Zang 2020,^94^ United States | SEER | 1999-2017 | 193,131  (46,383; 24%) | All ages | NR | ≥ 5 years | Urological | Urothelial bladder |
| Zaorsky 2017,^95^ United States | SEER | 1973-2012 | NR | All ages | NR | Range: 2 to >240 months | All cancer | All cancer |
| Zaorsky 2019,^96^ United States | SEER | 1992-2015 | 7,529,481  (3,661,011; 49%) | All ages | White: 82%  Black: 10%  Other: 7%  Unknown: 1% | Range: 2 to >120 months | All cancer (incl Gastrointestinal, Urological, Brain and other CNS, Breast, Gynaecological, Lung, Haematological, Skin, Head and neck, Prostate, Thyroid) | All cancer:  Anus, Bladder, Brain, Breast, Cervix, Colorectal, Oesophagus, Liver, Lung, Lymphoma, Melanoma, Oral cavity and pharynx, Pancreatic, Prostate, Stomach, Thyroid, Uterus |
| Zhai 2020,^97^ United States | SEER | 2014-2016 | 24,074 (NR) | All ages | White:  89%  Black:  6%  Asian:  5%  American Indian/ Alaska Native:  0.4% | Range: <1 to 3 years | Urological | Bladder, Non-metastatic (M0), within 3 years |
| Zhang 2021,^98^ United States | SEER | 1975-2016 | 6,356 (0; 0%) | ≥35;  65 years (median) | White: 80%  Black: 14%  Others: 5%  Unknown: 1% | Up to 42 years | Breast | Breast (Male) |
| Zheng 2022,^99^ United States | SEER | 2000-2018 | 63,560 (14,477; 23%) | All ages | White: 84%  Black: 11%  Asian: 0.5%  American Indian/ Alaska Native:  5% | Range: <1 to >10 years | Gastrointestinal | Esophagus |
| **Europe (1 Denmark, 1 Finland, 1 Germany, 3 Italy, 4 Netherlands, 4 Norway, 1 Spain, 3 Sweden, 1 Switzerland, 3 United Kingdom)** | | | | | | | | |
| Jensen 2008,^100^ Denmark | Cancer Registry Denmark, Death Register | 1978-2001 | 96,920  (48,210; 50%) | All ages | NR | Range: <1 to >10 years | Skin | Basal cell carcinoma,  Squamous cell carcinoma |
| Katuwal 2021,^101^ Finland | Cancer Registry Finland, Death Registry | 1971-2012 | 50,481  (50,481; 100%) | 25-74 | NR | Up to 12 years | Breast | Breast |
| Eisfeld 2023,^102^ North Rhine-Westphalia, Germany | Cancer Registry North Rhine-Westphalia, Germany | 2010-2019 | 14,815 (6,730; 45%) | All ages | NR | NR | Haematological | Multiple myeloma |
| Ascoli 2009,^103^ Italy | National mortality database | 1995-2002 | 946 (327; 35%) | ≥65  Range: 65-101 | NR | NR | Sarcoma | Classic (or ‘Mediterranean’) Kaposi’s sarcoma |
| Caocci 2020,^104^ Italy | Records from 19 Italian centres | 2012-2017 | 656 (284; 43%) | 18-89;  Mean: 53 | NR | 6 years (mean)  Range: 0.6-25.9 years | Haematological | Chronic myeloid leukaemia, treatment-specific (chronic phase treated with specific tyrosine kinase inhibitors) |
| Mangone 2021,^105^ Reggio Emilia Province, Italy | Cancer Registry Reggio Emilia Province including mortality data | 1996-2019 | 67,163 (32,245; 48%) | All ages | NR | Range: <2 to >10 years | All cancer | All cancer |
| de Vries 2021,^106^ Netherlands | Hospital, GP records and nationwide death registry from 7 centres | 1965-2018 | 4,919  (2,066; 42%) | ≥50;  Median: 27.8 | NR | 20.2 (median) Range: 0 to ≥ 40 years | Haematological | Hodgkin lymphoma |
| Hooning 2006,^107^ Netherlands | Hospital-based cancer registry of 2 centres | 1970-2000 | 7,425  (7,425; 100%) | <71;  Median: 50 | NR | 13.8 years (median)  Range: 1 to ≥ 25 years | Breast | Breast, Stage I-III, 1-year survivor |
| Otto 2006,^108^ Netherlands | European Randomized Study of Screening for Prostate Cancer participants, death registry | 1994-2002 | 2,211 (0; 0%) | 55-74;  Mean: 65.49  Median: 65.97 | NR | 12,610 person-years | Prostate | Prostate |
| van Monsjou 2016,^109^ Netherlands | Cancer Registry Netherlands and death registry | 1989-2008 | 14,393  (5,355; 37%) | All ages; Median: 61 | NR | 6.7 years (median)  Range:  <5 to ≥ 15 years | Head and neck | Squamous cell carcinoma of the oral cavity (OC) or oropharynx (OP) |
| Forster 2022,^110^ Norway | Cancer Registry Norway | 2013-2019 | 1,018 (0; 0%) | ≥40  Median: 71 | NR | NR | Prostate | Prostate |
| Fossa 2004,^111^ Norway | Cancer Registry Norway and death certificates | 1962-1997 | 3,378  (0; 0%) | ≤55 | NR | 41,960 person-years;  Follow up to 60-year-old | Testicular and other germ cell tumours | Malignant germ cell tumours |
| Hellesnes 2021,^112^ Norway | Cancer Registry Norway and death registry | 1980-2018 | 5,707  (0; 0%) | ≥16  Range: 16-83.8;  Median: 33.1 | NR | 18.7 years (median)  Range: 0-39 years | Testicular and other germ cell tumours | Testicular |
| Kvammen 2019,^113^ Norway | Cancer Registry Norway and death registry | 1953-2015 | 9,541  (0; 0%) | Median: <40 | NR | Range: 0 to 50 years | Testicular and other germ cell tumours | Testicular germ cell tumour |
| Ameijide 2019,^114^ Tarragona and Girona, Spain | Cancer Registry Tarragona and Girona, mortality registry and national index of death | 1985-2014 | 10,195 (10,195; 100%) | 15-84 | NR | 10, 20 years | Breast | Breast |
| Eriksson 2000,^115^ Sweden | Single institution data at Karolinska hospital and death register | 1972-1996 | 157  (60; 38%) | Median: 33  14-70 | NR | 16 years (median) Range: 0-23 years | Haematological | Hodgkin lymphoma, Stage I-III, treatment-specific (radiation therapy) |
| Van Hemelrijck 2010,^116^ Sweden | National prostate cancer registry and death register | 1997-2007 | 76,600  (0; 0%) | All ages | NR | 3.5-4.7 years (mean) | Prostate | Prostate, treatment-specific (endocrine therapy vs curative vs surveillance) |
| Zar 2008,^117^ Sweden | Cancer Registry Sweden and death registry | 1960-2001 | 4,884 (NR) | All ages | NR | Range: 0 to >1 year | Others, Gastrointestinal | Primary adenocarcinoma, Carcinoid in the small intestine |
| Levi 2002,^118^ Vaud, Switzerland | Cancer Registry (Vaud) and mortality statistics | 1974-1994 | 1,095  (1,095; 100%) | All ages | NR | ≥ 10 years | Breast | Breast, 10-year survivor |
| Henson 2016,^119^ England and Welsh, United Kingdom | National Statistics (England) and Cancer Registry (Welsh), and health and social care information on death data | 1971-2014 | 200,945 (124,279; 62%) | 15-39 | NR | ≥ 5 years | All cancer (incl Haematological, Urological, Sarcoma, Breast, Gynaecological, Brain and other CNS, Gastrointestinal, Head and neck, Lung, Skin, Others, Testicular and other germ cell tumours, Thyroid) | All cancer (except brain, bladder), 5-year survivors:  AML, Bladder, Bone tumour, Breast, Cervix, CNS tumours, Gastrointestinal, Head and neck, Hodgkin lymphoma, Kidney and genitourinary tract, Leukaemia (excl. AML), Lung, Melanoma, Non-Hodgkin lymphoma, Other, Ovary, Soft tissue sarcoma, Testicular, Thyroid |
| Swerdlow 2007,^120^ United Kingdom | Clinical databases of the British National Lymphoma Investigation, and 3 hospitals in UK | 1967-2000 | 7,033  (2,680; 38%) | All ages | NR | 11.1 years (mean) | Haematological | Hodgkin lymphoma |
| Wild 2007,^121^ Scotland, United Kingdom | National Health Service National Services Scotland and mortality record | 1981-2002 | 7,182  (7,182; 100%) | ≥30;  Mean: 65  Median: 65 | NR | 5 years (median) | Gynaecological | Endometrial |
| **Asia (1 China, 3 Japan, 4 South Korea)** | | | | | | | | |
| Li 2022,^122^ Shanghai, China | Cancer Registry (Shanghai Pudong), mortality registry | 2005-2020 | 1,088 (1,088; 100%) | All ages; Range: 20-99 | NR | NR | Gynaecological | Ovary |
| Gon 2023,^123^ Osaka, Japan | Cancer Registry (Osaka), neoplasms and other causes of death database | 1985-2013 | 688,473 (303,893; 44%) | All ages | NR | 4.1 years (median) | All cancer | All cancer |
| Harashima 2021,^124^ Japan | National Cancer Registry (Japan), National Death certificates | 2016 | 546,148  (243,989; 45%) | All ages | NR | Total: 6 months | All cancer | All cancer excl. CNS tumours |
| Kurisu 2022,^125^ Japan | National Cancer Registry (Japan) | 2016-2018 | 1,070,876 (479,922; 45%) | All ages | NR | Total: 2 years | All cancer (incl Urological, Breast, Gynaecological, Gastrointestinal, Head and neck, Haematological, Lung, Others, Prostate, Skin, Thyroid) | All cancer:  Bladder, Breast, Cervix, Corpus uteri, Colon, Gallbladder and other biliary tract, Head and neck, Kidney and urinary organs, Leukemia, Liver and intrahepatic bile ducts, Lung and bronchus, Lymphoma, Multiple myeloma, Oesophagus, Other, Ovary, Pancreas, Prostate, Rectum, Skin, Stomach, Thyroid |
| Jung 2023,^126^ South Korea | Cancer Registry South Korea, Cause of Death statistics | 2006-2017 | 40,890 (9,220; 23%) | All ages | NR | Up to 12 years | Head and neck | Head and neck |
| Kim 2022,^127^ South Korea | Cancer Registry South Korea, Cause of Death statistics | 2000-2017 | 231,388 (56,750; 25%) | ≥20;  Mean: 60.9 | NR | Range: 0 to 18 years | Gastrointestinal | Liver |
| Oh 2020,^128^ South Korea | Cancer Registry South Korea, Cause of Death statistics | 2000-2016 | 2,707,520 (1,296,720; 48%) | 20-85;  Mean: 59.3 | NR | Range: 0 to ≥10 years | All cancer (incl. Breast, Gastrointestinal, Lung, Prostate, Thyroid) | All cancer:  Breast, Colorectal, Lung, Prostate, Stomach, Thyroid |
| Shin 2010,^129^ South Korea | Cancer Registry South Korea , National Death data | 1993-2005 | 243,713 (146,954; 60%) | 20-79;  Mean: 51.5 | NR | 7.89 years (median) 8.26 years (mean)  Range: >=5 years | All cancer (incl. Breast, Gynaecological, Gastrointestinal, Lung, Prostate, Thyroid) | 8 cancers, 5-year survivor:  Breast, Cervix, Colorectal, Liver, Lung, Prostate, Stomach, Thyroid |
| **Australia/Oceania (4 Australia)** | | | | | | | | |
| Baade 2006,^130^ Queensland, Australia | Cancer Registry Queensland | 1982-2002 | 144,679 (68,144; 47%) | 20-79 | NR | Range: 0 to 20 years | All cancer (incl. Breast, Gynaecological, Gastrointestinal, Lung, Skin, Haematological, Prostate) | All cancer:  Breast, Cervical, Colorectal, Lung, Melanoma, Non-hodgkin lymphoma, Prostate |
| Beadle 2013,^131^ Australia | National Cancer Statistics, National Death Index | 1982-2005 | 179,653 (179,653; 100%) | 30-79 | NR | 6.3 years (mean) excluding 1^st^ year;  Range: 0-23 years | Breast | Breast, 1-year survivor |
| Koczwara 2021,^132^ South Australia, Australia | Cancer Registry South Australia, Death data | 1990-2016 | 32,646  (16,246; 50%) | All ages;  Mean: 60.3 | European: 93%  Asian:  0.6%  ATSI:  0.4%  Unknown: 6% | 17 years (median)  Range: 5 to 26 years | All cancer (incl. Breast, Gastrointestinal, Gynaecological, Head and neck, Haematological, Lung, Others, Prostate, Urological, Skin) | All cancer, 5-year survivor:  Breast, Gastrointestinal, Gynaecological, Head and neck, Lymphoma/ other haematological, Lung, Other/unknown, Prostate, Renal/ testicular/ bladder, Skin |
| Ye 2019,^133^ Tasmania, Australia | Cancer Registry Tasmania, Death Registry | 2006-2015 | 21,637  (9,481; 44%) | ≥15  66.5 (median); 65.8 (mean) | NR | 3.8 years (median)  4.1 years (mean)  Range: <1 to 9 years | All cancer (incl Breast, Gastrointestinal, Gynaecological, Head and neck, Haematological, Lung, Others, Prostate, Skin, Urological) | All cancer:  Breast, Colorectal, Digestive except colorectal, Female organs, Head and neck, Hematologic, Lung, Lymphoma, Other, Prostate, Skin, Urinary tract |
| **More than one country (1 North America, 1 North America + Europe, 1 US + UK)** | | | | | | | | |
| Fossa 2007,^134^ North America (US, Canada) and Europe (Denmark, Sweden, Norway, Finland) | Cancer Registry (Denmark, Sweden, Finland, Canada's Ontario, Norway), and SEER | 1943-2002 | 38,907  (0; 0%) | All ages | NR | 10 years (median)  Range: 1-55 years | Testicular and other germ cell tumours | Testicular, 1-year survivor |
| Jin 2021,^135^ Wales, United Kingdom and United States | Cancer Surveillance Data Wales, Mortality data | 2000-2015; | 3,624  (1,546, 43%) | ≥18;  Mean: 62.2 | NR | 6 months (median)  Range: <1 to ≥ 10 years | Brain and other CNS | CNS tumours |
|  | US SEER | 2005-2015 | 53,107 (23,224, 44%) | ≥18;  Mean: 58.8 | White: 76%  Black:  6%  Asian:  5%  American Indian/ Alaska Native:  0.5%  Hispanic:  12%  Unknown: 0.4% | 13 months (median)  Range: <1 to ≥ 10 years | Brain and other CNS | CNS tumours |
| Suh 2020,^136^ North America (US, Canada) | 27 academic institutions in North America | 1970-1999 | AYA: 5,804  (2,556,44%) | 15-20;  Median: 17 | White: 58%  Black: 3%  Other: 2%  Hispanic: 4%  Unknown: 33% | 21 years (median)  Range:  5 to >30years | All cancers (incl Sarcoma, Brain and other CNS, Haematological) | Selected cancers, 5-year survivor:  Bone, CNS tumours, Hodgkin lymphoma, Leukemias, Non-Hodgkin lymphoma, Soft tissue sarcoma |

**References**

1. Arora N, Talhouk A, McAlpine JN, Law MR, Hanley GE. Long-term mortality among women with epithelial ovarian cancer: a population-based study in British Columbia, Canada. BMC Cancer 2018;18:1039.

2. Abuamsha H, Kadri AN, Hernandez AV. Cardiovascular mortality among patients with non-Hodgkin lymphoma: differences according to lymphoma subtype. Hematol Oncol 2019;37:261-269.

3. Afifi AM, Saad AM, Al-Husseini MJ, Elmehrath AO, Northfelt DW, Sonbol MB. Causes of death after breast cancer diagnosis: a US population-based analysis. Cancer 2020;126:1559-1567.

4. Afifi AM, Elmehrath AO, Ruhban IA, et al. Causes of death following nonmetastatic colorectal cancer diagnosis in the U.S.: a population-based analysis. Oncologist 2021;26:733-739.

5. Afify AY, Ashry MH, Sadeq MA, Elsaid M. Causes of death after laryngeal cancer diagnosis: A US population-based study. Eur Arch Otorhinolaryngol 2023;280:1855-1864.

6. Agha A, Wang X, Wang M, et al. Long-term risk of death from heart disease among breast cancer patients. Front Cardiovasc Med 2022;9:784409.

7. Anderson C, Lund JL, Weaver MA, Wood WA, Olshan AF, Nichols HB. Noncancer mortality among adolescents and young adults with cancer. Cancer 2019;125:2107-2114.

8. Anderson C, Bae-Jump VL, Broaddus RR, Olshan AF, Nichols HB. Long-term patterns of excess mortality among endometrial cancer survivors. Cancer Epidemiol Biomarkers Prev 2021;30:1079-1088.

9. Appiah D, Farias RM, Olokede OA, et al. The influence of individual and neighborhood-level characteristics on rural-urban disparities in cardiovascular disease mortality among U.S. women diagnosed with breast and gynecologic cancers. Gynecol Oncol 2021;161:483-490.

10. Awad AK, Elgenidy A, Afifi AM, Sa MP, Ramlawi B. Specific causes of death among patients with cardiac sarcoma in the United States-An analysis of The Surveillance, Epidemiology, and End Results (SEER) Program. J Card Surg 2022;37:3961-3963.

11. Beard CJ, Travis LB, Chen MH, et al. Outcomes in stage I testicular seminoma: a population-based study of 9193 patients. Cancer 2013;119:2771-2777.

12. Chen J, Zheng Y, Wang H, et al. Cause of death among patients with colorectal cancer: a population-based study in the United States. Aging (Albany NY) 2020;12:22927-22948.

13. Chen L, Zhao X, Wang S. Factors leading to the risk of stroke mortality: a cross-sectional study with lung cancer patient-based large sample. Eur J Cancer Prev 2022;31:14-18.

14. Chen C, Xu F, Yuan S, et al. Competing risk analysis of cardiovascular death in patients with primary gallbladder cancer. Cancer Med 2023;12:2179-2186.

15. Chen B, Zhao X, Li X, Liu J, Tang J. Fatal heart disease in patients with bone and soft tissue sarcoma. Front Cardiovasc Med 2022;9.

16. Chen L, Zheng Y, Yu K, et al. Changing causes of death in persons with haematological cancers 1975-2016. Leukemia 2022;36:1850-1860.

17. Chen Y, He L, Lu X, et al. Causes of death among early-onset colorectal cancer population in the United States: a large population-based study. Front Oncol 2023;13:1094493.

18. Cheng J, Wang R, Wang X. Causes of death following endometrial cancer diagnosis: an analysis of 15 years of follow-up. J Biol Regul Homeost Agents 2022;36:565-573.

19. Chi K, Zhou R, Luo Z, et al. Non-cancer-specific survival in patients with primary central nervous system lymphoma: a multi-center cohort study. Front Oncol 2023;13:1096027.

20. Dai ZH, Tang M, Chen YL, et al. Incidence and risk factors for cerebrovascular-specific mortality in patients with colorectal cancer: a registry-based cohort study involving 563,298 Patients. Cancers 2022;14.

21. Dalal NH, Dores GM, Curtis RE, Linet MS, Morton LM. Cause-specific mortality in individuals with lymphoplasmacytic lymphoma/Waldenström macroglobulinaemia, 2000-2016. Br J Haematol 2020;189:1107-1118.

22. Dores GM, Curtis RE, Dalal NH, Linet MS, Morton LM. Cause-specific mortality following initial chemotherapy in a population-based cohort of patients with classical Hodgkin lymphoma, 2000-2016. J Clin Oncol 2020;38:4149-4162.

23. Du B, Wang F, Wu L, et al. Cause-specific mortality after diagnosis of thyroid cancer: a large population-based study. Endocrine 2021;72:179-189.

24. Eifler JB, Humphreys EB, Agro M, Partin AW, Trock BJ, Han M. Causes of death after radical prostatectomy at a large tertiary center. The Journal of urology 2012;188:798-801.

25. Elgenidy A, Afifi AM, Jalal PK. Survival and causes of death among patients with intrahepatic cholangiocarcinoma in the United States from 2000 to 2018. Cancer Epidemiol Biomarkers Prev 2022;31:2169-2176.

26. Elgenidy A, Awad AK, Cheema HA, et al. Cause-specific mortality among patients with renal cell carcinoma in the United States from 2000 to 2018. Urol Oncol 2023;41:209.e211-209.e220.

27. Elmehrath AO, Afifi AM, Al-Husseini MJ, et al. Causes of death among patients with metastatic prostate cancer in the US from 2000 to 2016. JAMA Netw Open 2021;4:e2119568.

28. Elmehrath AO, Afifi AM, Abdel-Malek R. Non-cancer death causes after ovarian cancer diagnosis: a population-based cohort. J Obstet Gynaecol Res 2021;47:1884-1891.

29. Fang F, Keating NL, Mucci LA, et al. Immediate risk of suicide and cardiovascular death after a prostate cancer diagnosis: cohort study in the United States. J Natl Cancer Inst 2010;102:307-314.

30. Felix AS, Bower JK, Pfeiffer RM, Raman SV, Cohn DE, Sherman ME. High cardiovascular disease mortality after endometrial cancer diagnosis: results from the Surveillance, Epidemiology, and End Results (SEER) Database. Int J Cancer 2017;140:555-564.

31. Feng Y, Jin H, Guo K, Wasan HS, Ruan S, Chen C. Causes of death after colorectal cancer diagnosis: a population-based study. Front Oncol 2021;11:647179.

32. Fung C, Fossa SD, Milano MT, Sahasrabudhe DM, Peterson DR, Travis LB. Cardiovascular disease mortality after chemotherapy or surgery for testicular nonseminoma: a population-based study. J Clin Oncol 2015;33:3105-3115.

33. Gad MM, Saad AM, Al-Husseini MJ, et al. Temporal trends, ethnic determinants, and short-term and long-term risk of cardiac death in cancer patients: a cohort study. Cardiovasc Pathol 2019;43:107147.

34. Gaitanidis A, Spathakis M, Tsalikidis C, Alevizakos M, Tsaroucha A, Pitiakoudis M. Risk factors for cardiovascular mortality in patients with colorectal cancer: a population-based study. Int J Clin Oncol 2019;24:501-507.

35. Guan T, Zhang H, Yang J, et al. Increased risk of cardiovascular death in breast cancer patients without chemotherapy or (and) radiotherapy: a large population-based study. Front Oncol 2021;10.

36. Guan T, Su M, Luo Z, et al. Long-term cardiovascular mortality among 80,042 older patients with bladder cancer. Cancers 2022;14.

37. Guan T, Jiang Y, Luo Z, et al. Long-term risks of cardiovascular death in a population-based cohort of 1,141,675 older patients with cancer. Age Ageing 2023;52.

38. Guo Y, Dong X, Yang F, et al. Effects of radiotherapy or radical prostatectomy on the risk of long-term heart-specific death in patients with prostate cancer. Front Oncol 2020;10:592746.

39. Guo Y, Dong X, Mao S, et al. Causes of death after prostate cancer diagnosis: a population-based study. Oxid Med Cell Longev 2022;2022:8145173.

40. Harvitkar RU, Peri H, Zallipalli SN, Joseph SJ, Gattupalli GB, Ansari K. Non-cancer causes of death in patients with pancreatic adenocarcinoma: a Surveillance, Epidemiology, and End Results (SEER)-based study. Cureus 2021;13:e20289.

41. Hisada M, Chen BE, Jaffe ES, Travis LB. Second cancer incidence and cause-specific mortality among 3104 patients with hairy cell leukemia: a population-based study. J Natl Cancer Inst 2007;99:215-222.

42. Howlader N, Mariotto AB, Besson C, et al. Cancer-specific mortality, cure fraction, and noncancer causes of death among diffuse large B-cell lymphoma patients in the immunochemotherapy era. Cancer 2017;123:3326-3334.

43. Lee CK, Aeppli D, Nierengarten ME. The need for long-term surveillance for patients treated with curative radiotherapy for Hodgkin's disease: University of Minnesota experience. Int J Radiat Oncol Biol Phys 2000;48:169-179.

44. Li G, Zhou Z, Yang W, et al. Long-term cardiac-specific mortality among 44,292 acute myeloid leukemia patients treated with chemotherapy: a population-based analysis. Journal of Cancer 2019;10:6161-6169.

45. Liu E, Guan X, Wei R, et al. Association between radiotherapy and death from cardiovascular disease among patients with cancer: a large population-based cohort study. J Am Heart Assoc 2022;11:e023802.

46. Liu G, Zhang BF. Age-specific cardiovascular disease-related mortality among patients with major gastrointestinal cancers: a SEER population-based study. Cancer Med 2023;12:17253-17265.

47. Lou T, Hu X, Lu N, Zhang T. Causes of death following gastric cancer diagnosis: a population-based analysis. Med Sci Monit 2023;29:e939848.

48. Low SK, Giannis D, Bahaie NS, Trong BLH, Moris D, Huy NT. Competing mortality in patients with neuroendocrine tumors. Am J Clin Oncol 2019;42:668-674.

49. Lu L, Ma L, Zhang X, Susanne Mullins C, Linnebacher M. Analyzing non-cancer causes of death of colorectal carcinoma patients in the US population for the years 2000-2016. Cancer Med 2021;10:2740-2751.

50. Lu Z, Teng Y, Ning X, Wang H, Feng W, Ou C. Long-term risk of cardiovascular disease mortality among classic Hodgkin lymphoma survivors. Cancer 2022;128:3330-3339.

51. Lyu Q, Nie Y, Yuan J, Wang D. Causes of death in female patients with bladder cancer after local tumor excision and radical cystectomy: a contemporary, US population-based analysis. Eur J Med Res 2022;27:230.

52. Massa ST, Osazuwa-Peters N, Christopher KM, et al. Competing causes of death in the head and neck cancer population. Oral oncology 2017;65:8-15.

53. Miao J, Wang Y, Gu X, et al. Risk of cardiovascular disease death in older malignant melanoma patients: a population-based study. Cancers 2022;14.

54. Moke DJ, Song Z, Liu L, Hamilton AS, Deapen D, Freyer DR. A population-based analysis of 30-year mortality among five-year survivors of adolescent and young adult cancer: the roles of primary cancer, subsequent malignancy, and other health conditions. Cancers 2021;13.

55. Peng FS, Wu WT, Zhang L, Shen JH, Yu DD, Mao LQ. Cause of death during upper tract urothelial carcinoma survivorship: a contemporary, population-based analysis. Front Oncol 2022;12:948289.

56. Sadeq MA, Ashry MH, Ghorab RMF, Afify AY. Causes of death among patients with cutaneous melanoma: a US population-based study. Sci Rep 2023;13:10257.

57. Shi T, Jiang C, Zhu C, Wu F, Fotjhadi I, Zarich S. Insurance disparity in cardiovascular mortality among non-elderly cancer survivors. Cardiooncology 2021;7:11.

58. Sonbol YT, Elgenidy A, Awad AK, et al. Stroke as a cause of death in patients with cancer: a SEER-based study. J Stroke Cerebrovasc Dis 2023;32:107154.

59. Song P, Lu N, Zhang J, et al. Cause of death of patients with non-muscular invasive, non-metastatic muscular invasive and metastatic bladder cancer after diagnosis. Am J Transl Res 2022;14:3494-3515.

60. Song P, Wu X, Yang L, et al. Second malignant tumors and non-tumor causes of death for patients with penile cancer during their survivorship. Cancer Control 2022;29:10732748221134789.

61. Stoltzfus KC, Zhang Y, Sturgeon K, et al. Fatal heart disease among cancer patients. Nat Commun 2020;11:2011.

62. Sturgeon KM, Deng L, Bluethmann SM, et al. A population-based study of cardiovascular disease mortality risk in US cancer patients. Eur Heart J 2019;40:3889-3897.

63. Su C, Wang Y, Wu F, Qiu Y, Tao J. Suicide and cardiovascular death among patients with multiple primary cancers in the United States. Front Cardiovasc Med 2022;9:857194.

64. Sun S, Wang W, He C. Cardiovascular mortality risk among patients with gastroenteropancreatic neuroendocrine neoplasms: a registry-based analysis. Oxid Med Cell Longev 2021;2021:9985814.

65. Udkoff J, Russell E, Knackstedt T, Holzer AM. Drivers of overall mortality in Merkel cell carcinoma: a population-based analysis. J Am Acad Dermatol 2023;88:1151-1152.

66. Vo JB, Ramin C, Barac A, Berrington de Gonzalez A, Veiga L. Trends in heart disease mortality among breast cancer survivors in the US, 1975–2017. Breast Cancer Res Treat 2022;192:611-622.

67. Vo JB, Ramin C, Lawrence WR, et al. Racial and ethnic disparities in treatment-related heart disease mortality among US breast cancer survivors. JNCI Cancer Spectr 2023;7.

68. Wang D, Yi L, Zhang L, Wang Z. Cause-specific mortality among patients with different molecular subtypes of T1-2N0M0 breast cancer: a population-based study. Medicine (Baltimore) 2021;100:e27605.

69. Wang Z, Wang J, Zhu Y, Liu C, Li X, Zeng X. Cause-specific mortality among survivors from T1N0M0 renal cell carcinoma: a registry-based cohort study. Front Oncol 2021;11:604724.

70. Wang Q, Zeng Z, Nan J, Zheng Y, Liu H. Cause of death among patients with thyroid cancer: a population-based study. Front Oncol 2022;12:852347.

71. Wang S, Ge C, Zhang J. Cardiovascular mortality risk in patients with bladder cancer: a population-based study. J Cardiovasc Dev Dis 2022;9.

72. Wang J, Wang Q, Du X, et al. Risk factors associated with cardiovascular mortality among gastric cancer patients: a population-based analysis. Jpn J Clin Oncol 2022;52:1365-1374.

73. Wang S, Ge C. High risk of non-cancer mortality in bladder cancer patients: evidence from SEER-Medicaid. J Cancer Res Clin Oncol 2023;149:10203-10215.

74. Weberpals J, Jansen L, Müller OJ, Brenner H. Long-term heart-specific mortality among 347 476 breast cancer patients treated with radiotherapy or chemotherapy: a registry-based cohort study. Eur Heart J 2018;39:3896-3903.

75. Weiner AB, Li EV, Desai AS, Press DJ, Schaeffer EM. Cause of death during prostate cancer survivorship: a contemporary, US population-based analysis. Cancer 2021;127:2895-2904.

76. Wu XQ, Li JY, Du WJ. Causes of death following small cell lung cancer diagnosis: a population-based analysis. BMC Pulm Med 2022;22:262.

77. Xia Y, Lin M, Huang J, Fan L. Cardiovascular disease related death among patients with esophagus cancer: a population-based competing risk analysis. Front Oncol 2022;12:976711.

78. Xie Z, Zhan X, Zheng Y, et al. High cardiovascular disease mortality after penile squamous cell carcinomas diagnosis: results from the United States SEER population, 2005-2016. Front Oncol 2022;12:1004791.

79. Xue Q, Che W, Xue L, Zhang X, Wang X, Lyu J. Causes of death in endometrial cancer survivors: a Surveillance, Epidemiology, and End Result-based analysis. Cancer Med 2023;12:10917-10930.

80. Yang F, Li C, Guo Y, et al. Effects of radical cystectomy, radiotherapy, and chemotherapy on the risk of long-term heart-specific death in bladder cancer patients. Transl Androl Urol 2021;10:3826-3836.

81. Yang P, Zheng Y, Chen J, et al. Immediate risk of non-cancer deaths after a cancer diagnosis. BMC Cancer 2021;21:963.

82. Yang Z, Leng K, Shi G. Causes of death among patients with hepatocellular carcinoma in United States from 2000 to 2018. Cancer Med 2023;12:13076-13085.

83. Yang L, Wu X, Zhou J, et al. Second malignant tumors and non-tumor causes of death for patients with localized and regional kidney cancer after diagnosis. Eur J Med Res 2023;28:206.

84. Yao H, Shi H, Fan M, Yuan L, Lin R. Cardiovascular-specific mortality among gastrointestinal stromal tumor patients: a population-based analysis. Oxid Med Cell Longev 2023;2023:3619306.

85. Ye Y, Zheng Y, Miao Q, Ruan H, Zhang X. Causes of death among prostate cancer patients aged 40 years and older in the United States. Front Oncol 2022;12:914875.

86. Yin J, Zhao M, Lu T, et al. Non-lung cancer specific mortality after lobectomy or sublobectomy in patients with stage IA non-small cell lung cancer ≤2 cm: a propensity score analysis. J Surg Oncol 2019;120:1486-1496.

87. Yin X, Fan F, Zhang B, Hu Y, Sun C. Cardiovascular-specific mortality among multiple myeloma patients: a population-based study. Ther Adv Hematol 2022;13:20406207221086755.

88. Yin X, You L, Hu X. Role of radiation therapy in mortality among adolescents and young adults with lymphoma: differences according to cause of death. Cancers 2022;14.

89. Yin XJ, Wang R, Shen HS, Jin J, Zhu HH. At what point are long-term (>5 Years) survivors of APL safe? a study from the SEER database. Cancers 2023;15.

90. Youn P, Milano MT, Constine LS, Travis LB. Long-term cause-specific mortality in survivors of adolescent and young adult bone and soft tissue sarcoma: a population-based study of 28,844 patients. Cancer 2014;120:2334-2342.

91. Yu GP, Mehta V, Branovan D, Huang Q, Schantz SP. Non-cancer-related deaths from suicide, cardiovascular disease, and pneumonia in patients with oral cavity and oropharyngeal squamous carcinoma. Arch Otolaryngol Head Neck Surg 2012;138:25-32.

92. Yu DD, Chen WK, Wu CY, et al. Cause of death during renal cell carcinoma survivorship: a contemporary, population-based analysis. Front Oncol 2022;12:864132.

93. Zagars GK, Ballo MT, Lee AK, Strom SS. Mortality after cure of testicular seminoma. J Clin Oncol 2004;22:640-647.

94. Zang Y, Li X, Cheng Y, Qi F, Yang N. An overview of patients with urothelial bladder cancer over the past two decades: a Surveillance, Epidemiology, and End Results (SEER) study. Ann Transl Med 2020;8:1587.

95. Zaorsky NG, Churilla TM, Egleston BL, et al. Causes of death among cancer patients. Ann Oncol 2017;28:400-407.

96. Zaorsky NG, Zhang Y, Tchelebi LT, Mackley HB, Chinchilli VM, Zacharia BE. Stroke among cancer patients. Nat Commun 2019;10:5172.

97. Zhai M, Tang C, Li M, et al. Short-term mortality risks among patients with non-metastatic bladder cancer. BMC Cancer 2020;20:1148.

98. Zhang H, Lin W, Chen D, et al. Cardiovascular and other competing causes of death in male breast cancer patients: a population-based epidemiologic study. Clin Interv Aging 2021;16:1393-1401.

99. Zheng X, Zhang A, Xiao Y, et al. What causes death in esophageal cancer patients other than the cancer itself: a large population-based analysis. Journal of Cancer 2022;13:3485-3494.

100. Jensen A, Bautz A, Olesen AB, Karagas MR, Sørensen HT, Friis S. Mortality in Danish patients with nonmelanoma skin cancer, 1978-2001. The British journal of dermatology 2008;159:419-425.

101. Katuwal S, Jousilahti P, Pukkala E. Causes of death among women with breast cancer: a follow-up study of 50 481 women with breast cancer in Finland. Int J Cancer 2021;149:839-845.

102. Eisfeld C, Kajüter H, Möller L, Wellmann I, Shumilov E, Stang A. Time trends in survival and causes of death in multiple myeloma: a population-based study from Germany. BMC Cancer 2023;23:317.

103. Ascoli V, Minelli G, Kanieff M, Crialesi R, Frova L, Conti S. Cause-specific mortality in classic Kaposi's sarcoma: a population-based study in Italy (1995-2002). Br J Cancer 2009;101:1085-1090.

104. Caocci G, Mulas O, Annunziata M, et al. Long-term mortality rate for cardiovascular disease in 656 chronic myeloid leukaemia patients treated with second- and third-generation tyrosine kinase inhibitors. Int J Cardiol 2020;301:163-166.

105. Mangone L, Mancuso P, Tarantini L, et al. A population-based study of cardiovascular disease mortality in Italian cancer patients. Cancers 2021;13.

106. de Vries S, Schaapveld M, Janus CPM, et al. Long-term cause-specific mortality in Hodgkin lymphoma patients. J Natl Cancer Inst 2021;113:760-769.

107. Hooning MJ, Aleman BM, van Rosmalen AJ, Kuenen MA, Klijn JG, van Leeuwen FE. Cause-specific mortality in long-term survivors of breast cancer: a 25-year follow-up study. Int J Radiat Oncol Biol Phys 2006;64:1081-1091.

108. Otto SJ, Schröder FH, de Koning HJ. Risk of cardiovascular mortality in prostate cancer patients in the Rotterdam randomized screening trial. J Clin Oncol 2006;24:4184-4189.

109. van Monsjou HS, Schaapveld M, Hamming-Vrieze O, de Boer JP, van den Brekel MW, Balm AJ. Cause-specific excess mortality in patients treated for cancer of the oral cavity and oropharynx: a population-based study. Oral oncology 2016;52:37-44.

110. Forster RB, Kjellstadli C, Myklebust T, et al. Treatment and 30-day mortality after myocardial infarction in prostate cancer patients: a population-based study from Norway. Cardiology 2023;148:83-92.

111. Fosså SD, Aass N, Harvei S, Tretli S. Increased mortality rates in young and middle-aged patients with malignant germ cell tumours. Br J Cancer 2004;90:607-612.

112. Hellesnes R, Myklebust T, Fosså SD, et al. Testicular cancer in the cisplatin era: causes of death and mortality rates in a population-based cohort. J Clin Oncol 2021;39:3561-3573.

113. Kvammen Ø, Myklebust T, Solberg A, et al. Causes of inferior relative survival after testicular germ cell tumor diagnosed 1953-2015: a population-based prospective cohort study. PLoS One 2019;14:e0225942.

114. Ameijide A, Clèries R, Carulla M, et al. Cause-specific mortality after a breast cancer diagnosis: a cohort study of 10,195 women in Girona and Tarragona. Clin Transl Oncol 2019;21:1014-1025.

115. Eriksson F, Gagliardi G, Liedberg A, et al. Long-term cardiac mortality following radiation therapy for Hodgkin's disease: analysis with the relative seriality model. Radiother Oncol 2000;55:153-162.

116. Van Hemelrijck M, Garmo H, Holmberg L, et al. Absolute and relative risk of cardiovascular disease in men with prostate cancer: results from the Population-Based PCBaSe Sweden. J Clin Oncol 2010;28:3448-3456.

117. Zar N, Garmo H, Holmberg L, Hellman P. Risk of second primary malignancies and causes of death in patients with adenocarcinoma and carcinoid of the small intestine. Eur J Cancer 2008;44:718-725.

118. Levi F, Randimbison L, Te VC, La Vecchia C. Long-term mortality of women with a diagnosis of breast cancer. Oncology 2002;63:266-269.

119. Henson KE, Reulen RC, Winter DL, et al. Cardiac mortality among 200 000 five-year survivors of cancer diagnosed at 15 to 39 years of age: the teenage and young adult cancer survivor study. Circulation 2016;134:1519-1531.

120. Swerdlow AJ, Higgins CD, Smith P, et al. Myocardial infarction mortality risk after treatment for Hodgkin disease: a collaborative British cohort study. J Natl Cancer Inst 2007;99:206-214.

121. Wild SH, Bryden JR, Lee RJ, et al. Cancer, cardiovascular disease and diabetes mortality among women with a history of endometrial cancer. Br J Cancer 2007;96:1747-1749.

122. Li X, Zhang M, Chen Y, Lv H, Du Y. Longitudinal analysis of ovarian cancer death patterns during a rapid transition period (2005-2020) in Shanghai, China: a population-based study. Front Oncol 2022;12:1003297.

123. Gon Y, Zha L, Sasaki T, et al. Stroke mortality in cancer survivors: a population-based study in Japan. Thromb Res 2023;222:140-148.

124. Harashima S, Fujimori M, Akechi T, et al. Death by suicide, other externally caused injuries and cardiovascular diseases within 6 months of cancer diagnosis (J-SUPPORT 1902). Jpn J Clin Oncol 2021;51:744-752.

125. Kurisu K, Fujimori M, Harashima S, et al. Suicide, other externally caused injuries, and cardiovascular disease within 2 years after cancer diagnosis: a nationwide population-based study in Japan (J-SUPPORT 1902). Cancer Med 2023;12:3442-3451.

126. Jung YS, Lee D, Jung KW, Cho H. Long-term survivorship and non-cancer competing mortality in head and neck cancer: a nationwide population-based study in South Korea. Cancer Res Treat 2023;55:50-60.

127. Kim BH, Lee D, Jung KW, Won YJ, Cho H. Cause of death and cause-specific mortality for primary liver cancer in South Korea: a nationwide population-based study in hepatitis B virus-endemic area. Clin Mol Hepatol 2022;28:242-253.

128. Oh CM, Lee D, Kong HJ, et al. Causes of death among cancer patients in the era of cancer survivorship in Korea: attention to the suicide and cardiovascular mortality. Cancer Med 2020;9:1741-1752.

129. Shin DW, Ahn E, Kim H, Park S, Kim YA, Yun YH. Non-cancer mortality among long-term survivors of adult cancer in Korea: national cancer registry study. Cancer Causes Control 2010;21:919-929.

130. Baade PD, Fritschi L, Eakin EG. Non-cancer mortality among people diagnosed with cancer (Australia). Cancer Causes Control 2006;17:287-297.

131. Beadle GF, McCarthy NJ, Baade PD. Effect of age at diagnosis of breast cancer on the patterns and risk of mortality from all causes: a population-based study in Australia. Asia-Pac J Clin Oncol 2013;9:129-138.

132. Koczwara B, Meng R, Miller MD, et al. Late mortality in people with cancer: a population-based Australian study. Med J Aust 2021;214:318-323.

133. Ye Y, Otahal P, Marwick TH, Wills KE, Neil AL, Venn AJ. Cardiovascular and other competing causes of death among patients with cancer from 2006 to 2015: an Australian population-based study. Cancer 2019;125:442-452.

134. Fosså SD, Gilbert E, Dores GM, et al. Noncancer causes of death in survivors of testicular cancer. J Natl Cancer Inst 2007;99:533-544.

135. Jin K, Brennan PM, Poon MTC, Sudlow CLM, Figueroa JD. Raised cardiovascular disease mortality after central nervous system tumor diagnosis: analysis of 171,926 patients from UK and USA. Neurooncol Adv 2021;3:vdab136.

136. Suh E, Stratton KL, Leisenring WM, et al. Late mortality and chronic health conditions in long-term survivors of early-adolescent and young adult cancers: a retrospective cohort analysis from the Childhood Cancer Survivor Study. Lancet Oncol 2020;21:421-435.
