# Supplementary material for: Cardiovascular mortality in people with cancer compared to the general population: A systematic review and meta‐analysis
Source: Cancer Med. 2024 Aug 3;13(15):e70057. doi: 10.1002/cam4.70057 (PMC11297437; doi:10.1002/cam4.70057)
Supplement: Supplementary file 3 — Table S2. [file CAM4-13-e70057-s001.docx]

**Supplementary Table 2** Summary of studies reporting on SMRs by CVD and cancer type. Figures represent SMR extracted from the original studies

| **Cancer types** | **Type of cardiovascular diseases** | | | | | | | | |
| --- | --- | --- | --- | --- | --- | --- | --- | --- | --- |
|  | **Diseases of circulatory system/ cardiovascular diseases** | **Diseases of heart** | **Ischemic heart disease** | **Cardiomyopathy/ congestive heart failure/heart failure** | **Hypertension** | **Atherosclerosis** | **Aneurysm and dissection** | **Other** | **Cerebrovascular/ stroke** |
| Cancers combined    TOTAL: 52  Lower: 9  Non-significant: 4  Higher: 39 | 0.61 (0.58–0.65)  0.90 (0.88–0.93)  0.97 (0.90-1.04)  1.13 (1.13–1.14)  1.19 (1.17-1.21)  1.38 (1.33-1.43)  1.38 (1.33-1.44)  1.4 (1.3-1.4)  1.42 (1.38-1.47)  1.55 (1.50-1.60)  1.65 (1.63–1.67)  2.02 (2.01–2.04)  2.13 (2.13-2.14)  4.4 (3.7-5.2) | 0.68 (0.57-0.82)  0.90 (0.88-0.93)  0.96 (0.92-1.00)  1.16 (1.15-1.16)  1.22 (1.04-1.43)  1.58 (1.52-1.64)  2.06 (2.05–2.07)  2.13 (2.12-2.14)  2.24 (2.23-2.25)  2.36 (2.35-2.37) | 0.65 (0.58–0.73)  1.30 (1.17–1.45)  1.3 (1.2-1.4) | 1.5 (1.3-1.7) | 0.57 (0.45–0.70)  1.32 (1.04-1.65)  2.33 (2.25–2.42) | 1.95 (1.19-3.01)  2.17 (2.06–2.29) | 1.01 (0.71-1.39)  1.76 (1.67–1.85) | 1.77 (1.30-2.37)  2.06 (1.95–2.18)  2.45 (2.38-2.53)  2.9 (2.4-3.5)  3.99 (3.07–5.09) | 0.59 (0.55–0.64)  0.73 (0.70-0.75)  0.83 (0.80-0.87)  1.15 (0.99–1.34)  1.05 (1.04-1.06)  1.44 (1.31-1.57)  1.75 (1.71-1.80)  1.83 (1.80–1.86)  2.07 (1.86–2.30)  2.17 (2.15-2.19)  2.18 (2.16-2.20)  2.21 (2.19-2.23) |
| Breast  TOTAL: 67  Lower: 18  Non-significant: 26  Higher: 23 | 0.66 (0.51-0.83)  0.75 (0.73-0.77)  0.89 (0.69-1.14)  0.96 (0.86-1.05)  0.99 (0.91-1.08)  1.2 (1.0-1.5)  1.06 (1.05–1.07)  1.12 (1.01-1.24)  1.17 (1.14-1.20)  1.2 (1.1-1.4)  1.22 (1.10-1.36)  1.25 (1.15-1.36)  1.38 (1.25-1.51)  1.41 (1.37–1.45)  2.20 (2.15-2.25)  3.78 (3.58–4.00)  4.27 (3.05–5.82)  4.78 (3.88–5.84)  4.95 (4.20–5.80)  6.38 (6.32–6.45)  6.58 (6.14–7.05) | 0.74 (0.69-0.79)  0.80 (0.76-0.83)  0.82 (0.80-0.85)  0.84 (0.79-0.90)  0.87 (0.85-0.89)  0.89 (0.87-0.91)  0.90 (0.88-0.92)  0.91 (0.90-0.93)  0.94 (0.92-0.95)  0.91 (0.79-1.02)  0.93 (0.81-1.06)  0.95 (0.44-1.81)  1.00 (0.98-1.02)  1.01 (0.98-1.03)  1.04 (0.92-1.17)  1.31 (1.22-1.42)  1.58 (1.24–1.98)  1.99 (1.97-2.02)  5.85 (5.32-6.41)  6.57 (5.60-7.66) | 0.76 (0.74-0.79)  0.79 (0.45-1.28)  0.83 (0.59-1.13)  1.05 (0.74–1.44)  1.2 (1.0-1.4)  1.21 (1.17-1.25) | 1.0 (0.7-1.6)  1.20 (0.94–1.51) | 0.58 (0.30-1.01)  0.80 (0.48–1.26)  0.98 (0.93-1.03) | 0.84 (0.43–1.47)  0.95 (0.86-1.05) | 0.89 (0.80-0.99) | 0.75 (0.67-0.84)  0.88 (0.32–1.93)  1.16 (0.58–2.08)  1.5 (0.8-2.8) | 0.67 (0.49-0.89)  0.73 (0.70-0.76)  0.87 (0.85-0.89)  0.89 (0.87-0.91)  0.84 (0.68-1.03)  1.15 (0.95–1.37)  1.07 (1.01-1.12)  1.94 (1.9-1.99) |
| Gastrointestinal (e.g., colorectal, gastric, pancreatic, liver)  TOTAL: 143  Lower: 17  Non-significant: 23  Higher: 103 | 0.39 (0.26-0.55)  0.51 (0.43-0.59)  0.55 (0.50-0.61)  0.9 (0.7-1.1)  1.0 (0.5–2.1)  1.03 (0.88-1.21)  1.07 (0.74-1.55)  1.15 (1.09–1.20)  1.15 (1.14-1.16)  1.16 (1.08–1.26)  1.17 (1.09-1.26)  1.21 (1.07–1.37)  1.27 (1.20-1.36)  1.31 (1.25–1.37)  1.34 (1.20–1.50)  1.35 (1.14-1.59)  1.41 (1.25–1.58)  1.43 (1.39–1.48)  1.44 (1.33–1.57)  1.66 (1.61–1.70)  1.78 (1.49–2.12)  1.87 (1.86 -1.88)  1.88 (1.66–2.13)  1.94 (1.62-2.29)  2.11 (2.01-2.20)  2.14 (1.90–2.42)  2.26 (2.04–2.50)  2.27 (2.08–2.46)  2.28 (2.19-2.38)  2.38 (2.11–2.67)  3.23 (2.97-3.52)  3.35 (3.24-3.47)  4.24 (4.04-4.44)  11.7 (11.3-12)  15.84 (15.83-15.84) | 0.49 (0.26-0.86)  0.68 (0.48-0.94)  0.74 (0.69-0.80)  0.80 (0.72-0.89)  0.8 (0.8-0.9)  0.92 (0.87-0.98)  0.30 (0.04-1.09)  1.15 (1.14–1.17)  1.15 (1.14–1.17)  1.19 (1.10-1.28)  1.25 (1.23-1.27)  1.38 (1.33-1.44)  1.87 (1.85 -1.88)  2.06 (2.03-2.08)  2.21 (2.12-2.30)  2.24 (2.13-2.35)  2.36 (2.25-2.47)  2.58 (2.45-2.72)  2.79 (2.6-2.98)  3.21 (2.89-3.56)  3.27 (3.23-3.31)  3.38 (3.25-3.51) | 0.11 (0.01-0.41)  0.46 (0.36-0.59)  0.62 (0.45-0.85)  0.8 (0.6-1.1) | 1.3 (0.7-2.5) | 0.49 (0.30-0.75)  0.21 (0.01-1.18)  0.58 (0.30-1.01)  1.07 (0.66-1.64)  1.17 (1.09–1.25)  1.20 (1.13–1.26)  1.63 (1.29-2.03)  1.74 (1.27-2.32)  2.13 (1.71-2.62)  2.54 (2.06-3.10)  2.77 (2.62-2.93)  2.83 (2.73 -2.94)  2.94 (1.79-4.53)  2.94 (2.07-4.05) | 0.26 (0.01-1.42)  1.15 (1.02–1.29)  1.17 (1.06–1.29)  1.42 (1.05-1.89)  1.92 (1.83 -2.00)  2.39 (1.46-3.69)  2.50 (1.71-3.54)  2.79 (2.52-3.08)  3.46 (2.54-4.60)  3.84 (2.2-6.24) | 0.54 (0.20-1.18)  0.97 (0.88-1.07)  1.02 (0.91-1.14)  1.24 (0.88-1.70)  1.48 (1.41-1.56)  1.68 (1.07-2.52)  1.91 (1.36-2.61)  2.16 (1.15-3.69)  3.58 (1.54-7.04)  3.91 (3.53-4.33)  5.05 (3.52-7.02) | 1.49 (0.74-2.67)  1.5 (0.5-4.7)  1.14 (1.03–1.26)  1.15 (1.02–1.3)  1.80 (1.25-2.50)  1.84 (1.09-2.91)  1.91 (1.28-2.74)  1.97 (1.85-2.08)  3.06 (1.67-5.13)  3.10 (2.0-4.8)  3.32 (2.28-4.66)  3.40 (3.07-3.75)  10.35 (2.82-26.49) | 0.45 (0.36-0.55)  0.49 (0.32-0.73)  0.57 (0.50-0.64)  0.7 (0.7-0.8)  0.9 (0.7–1.2)  0.99 (0.90-1.09)  1.02 (0.99–1.06)  1.03 (1–1.06)  1.15 (0.99-1.33)  1.19 (0.99-1.42)  1.08 (1.06-1.11)  1.41 (1.29-1.53)  1.49 (1.39-1.6)  1.56 (1.35-1.78)  1.70 (1.53-1.88)  1.72 (1.53-1.92)  1.83 (1.81 -1.86)  2.08 (2.02-2.14)  2.10 (1.93-2.28)  2.13 (1.45-3.02)  2.14 (1.95-2.35)  2.56 (2.06-3.15)  3.11 (2.71-3.56)  3.28 (2.62-4.04)  3.30 (3.22-3.39)  3.33 (3.06-3.62)  3.37 (3.08-3.69)  6.07 (5.15-7.1)  7.21 (6.31-8.21)  9.19 (7.81-10.73)  26.32 (25.03-27.64)  36.85 (35.50-38.22)  62.29 (61.06-63.54) |
| Prostate  TOTAL: 64  Lower: 31  Non-significant: 12  Higher: 21 | 0.37 (0.18-0.68)  0.46 (0.29-0.71)  0.49 (0.36-0.64)  0.71 (0.68-0.74)  0.73 (0.62-0.85)  0.88 (0.83–0.93)  0.99 (0.99-1.00)  1.16 (0.91-1.46)  1.09 (1.06-1.12)  1.28 (1.24–1.32)  1.34 (1.26-1.42)  1.36 (1.35-1.37)  1.55 (1.47-1.64) | 0.27 (0.24–0.31)  0.44 (0.20-0.85)  0.55 (0.51-0.59)  0.69 (0.63-0.75)  0.76 (0.75-0.77)  0.76 (0.75-0.77)  0.81 (0.81-0.82)  0.98 (0.94-1.01)  1.32 (1.32-1.33)  1.48 (1.41-1.54)  1.77 (1.75-1.78) | 0.45 (0.26-0.74)  0.45 (0.28-0.69)  0.73 (0.65-0.80)  0.74 (0.65-0.85)  0.77 (0.61-0.97)  0.72 (0.29-1.48)  0.96 (0.89-1.03)  0.97 (0.92-1.03)  1.21 (1.16-1.26)  1.25 (1.18-1.32) | 0.85 (0.72-0.99)  1.05 (0.75-1.45)  1.24 (1.12-1.37) | 0.49 (0.30-0.75)  0.87 (0.83-0.91)  1.08 (1.03-1.13)  1.69 (1.35-2.08)  2.23 (2.16-2.30) | 0.82 (0.75-0.89)  1.67 (1.61-1.74) | 0.66 (0.62-0.71)  0.95 (0.91-0.99) | 0.74 (0.68-0.81)  0.83 (0.68-1.00)  0.87 (0.58-1.25)  1.01 (0.87-1.16)  1.29 (1.23-1.36) | 0.32 (0.23–0.42)  0.39 (0.19-0.69)  0.49 (0.21-0.98)  0.76 (0.74-0.78)  0.80 (0.79-0.82)  0.82 (0.81-0.84)  0.99 (0.81-1.19)  1.01 (0.90-1.13)  1.24 (1.14-1.35)  1.31 (1.13-1.50)  1.33 (1.19-1.48)  1.51 (1.49-1.53)  1.72 (1.69-1.76) |
| Lung  TOTAL: 26  Lower: 2  Non-significant: 8  Higher: 16 | 0.55 (0.42-0.70)  0.86 (0.65-1.07)  0.92 (0.78-1.06)  1.33 (1.26–1.41)  1.7 (1.2-2.4)  1.84 (1.39-2.44)  2.04 (2.02–2.07)  2.17 (2.11–2.24)  3.27 (2.66-4.02)  3.40 (3.18-3.62)  3.79 (3.74-3.85) | 0.47 (0.13-1.21)  1.03 (0.84-1.22)  1.10 (0.99-1.21)  2.30 (2.27-2.34)  5.71 (5.61-5.81) | 1.03 (0.66-1.53)  1.6 (1.1-2.3) | 2.6 (1.0-6.8) | 0.29 (0.04-1.06) |  |  | 4.8 (1.2-19.0) | 0.42 (0.28-0.61)  1.70 (1.65-1.75)  1.73 (1.69-1.78)  2.76 (2.34-3.24)  4.98 (4.77-5.21) |
| Hematological cancer (e.g., lymphoma, leukemia)  TOTAL: 146  Lower: 2  Non-significant: 21  Higher: 123 | 0.67 (0.56-0.80)  1 (0.67-1.42)  1.1 (1.0-1.1)  1.27 (0.86-1.86)  1.6 (1.0-2.4)  1.21 (1.08–1.34)  1.29 (1.07-1.54)  1.31 (1.06–1.61)  1.35 (1.25-1.44)  1.44 (1.32–1.57)  1.53 (1.17-2.00)  1.54 (1.30-1.80)  1.57 (1.50–1.65)  1.64 (1.46-1.84)  1.7 (1.5-2.1)  1.70 (1.66-1.73)  1.84 (1.78–1.89)  1.89 (1.60–2.22)  1.89 (1.86-1.91)  1.9 (1.7-2.1)  1.97 (1.58–2.46)  2.01 (1.86-2.18)  2.05 (1.79-2.34)  2.08 (2.06-2.10)  2.18 (2.01–2.36)  2.24 (1.67-2.93)  2.42 (1.82-3.16)  2.52 (2.18–2.91)  2.54 (2.49-2.59)  2.55 (2.00-3.21)  2.7 (1.6-4.4)  2.8 (2.28-3.42)  2.89 (2.78-3.01)  2.9 (1.6-5.2)  3.06 (2.52-3.69)  3.3 (1.8-6.0)  3.68 (3.54-3.83)  3.8 (3.5-4.2)  3.87 (3.42–4.37)  3.89 (3.71-4.08)  4.22 (3.78-4.72)  4.26 (3.91-4.63)  4.44 (4.10-4.81)  5.5 (5.0-6.1)  6.35 (5.89-6.82)  7.2 (4.3-11.5)  8.1 (6.6-9.9)  8.9 (7.8-10.1)  11.32 (10.77-11.89)  13.37 (12.93-13.81)  20.7 (20.07-21.38) | 1.0 (0.9-1.1)  1.25 (1.22-1.28)  1.56 (1.52-1.61) 1.75 (1.71-1.79)  1.9 (1.7-2.1)  1.92 (1.90-1.95)  1.94 (1.88–2.01)  2.14 (1.51-2.94) 2.14 (2.12-2.16)  2.24 (1.65-2.95)  2.66 (2.60-2.72)  2.71 (2.64-2.77)  2.96 (2.83-3.10)  3.04 (2.94-3.13)  3.96 (3.79-4.13)  3.98 (3.49-4.53)  4.06 (3.86-4.28)  4.24 (3.92-4.59)  5.47 (5.26-5.68) | 1.4 (0.9-2.3)  1.8 (0.9-3.6)  1.47 (1.35-1.60)  1.5 (1.3-1.9)  2.5 (2.1-2.9)  3.4 (3.1-3.8)  3.7 (3.0-4.5)  4.7 (3.2-6.7)  5.0 (2.7-8.6) | 1.8 (0.6-5.6)  2.7 (1.7-4.2)  3.5 (2.6-4.7)  8.2 (3.7-18.3) | 1.2 (0.8-1.9)  3.67 (0.76-10.72)  2.00 (1.74-2.29)  2.31 (1.92–2.77)  2.50 (2.31-2.71)  2.89 (2.74-3.05)  3.98 (3.12-5.07)  3.98 (2.98-5.31)  4.59 (3.38-6.23)  4.68 (4.21-5.19)  5.03 (1.63-11.73)  5.17 (2.46-10.8) | 1.64 (0.88-3.05)  1.71 (1.46-2.01)  1.89 (1.69-2.10)  1.98 (1.57–2.46)  2.04 (1.90-2.20)  2.44 (1.56-3.83)  2.45 (2.04-2.94)  2.5 (1.4-4.2)  3.48 (1.12-10.8)  3.56 (2.65-4.79) | 1.11 (0.80–1.51)  1.15 (0.96-1.39)  1.19 (0.93-1.51)  1.51 (0.86-2.66)  1.27 (1.13-1.43)  1.30 (1.19-1.41)  1.94 (1.30-2.90)  2.04 (1.41-2.96)  3.19 (1.20-8.49) | 1.19 (0.80–1.71)  2.5 (0.9-6.6)  7.4 (1.0-52.6)  1.61 (1.31-1.98)  1.91 (1.69-2.17)  2.02 (1.60-2.55)  2.09 (1.92-2.28)  3.13 (2.06-4.76)  4.06 (1.31-12.6)  4.91 (3.26-7.39)  5.34 (3.97-7.17)  14.5 (10.6-19.9) | 0.61 (0.38-0.93)  0.9 (0.7-1.2)  0.97 (0.93-1.01)  1.00 (0.81-1.21)  1.18 (1.12-1.24)  1.31 (1.21-1.4)  1.38 (1.26–1.49)  1.47 (1.39-1.55)  1.5 (1.1-2.0)  1.71 (1.65-1.77)  1.77 (1.73-1.81)  1.87 (1.76-1.99)  2.29 (2.05-2.57) 2.55 (2.25-2.88)  2.55 (2.42-2.69)  2.82 (1.41-5.05)  2.9 (1.5-3.3)  3.36 (2.96-3.81)  4.00 (2.41-6.24)  5.56 (4.34-7.12) |
| Testicular and other germ cell tumours  TOTAL: 42  Lower: 1  Non-significant: 35  Higher: 6 | 0.81 (0.60-1.07)  0.85 (0.49-1.46)  0.91 (0.80-1.05)  0.96 (0.85-1.09)  0.96 (0.91-1.02)  0.98 (0.94-1.04)  1.0 (0.9-1.1)  1.01 (0.86-1.18)  1.01 (0.93–1.09)  1.03 (0.91-1.15)  1.08 (0.97-1.21)  1.09 (0.99-1.22)  1.2 (1.0-1.5)  1.33 (0.80-2.21)  1.36 (1.03-1.78) | 0.83 (0.60-1.12)  0.95 (0.90-1.02)  0.97 (0.92-1.03)  1.03 (0.90-1.17)  1.25 (0.90-1.70)  1.37 (0.97-1.95)  1.61 (1.21-2.24)  8.29 (7.12-9.6) | 0.90 (0.84-0.98)  0.93 (0.88-1.00)  1.0 (0.9-1.1)  1.05 (0.92-1.22)  1.1 (0.9-1.4)  1.12 (0.91-1.37) | 1.1 (0.7-1.5) | 1.32 (0.90-1.87)  1.66 (0.83-2.98)  1.39 (1.01-1.89) |  |  | 0.95 (0.84-1.09)  0.98 (0.88-1.11)  1.11 (0.86-1.42)  6.51 (0.79-23.53)  2.4 (1.4-4.1) | 0.65 (0.40-1.05)  0.93 (0.64-1.35)  1.07 (0.43-2.20)  2.40 (1.15-4.42) |
| Gynaecological (e.g., ovarian, endometrial, cervix)  TOTAL: 64  Lower: 4  Non-significant: 30  Higher: 30 | 0.75 (0.63-0.89)  0.71 (0.41-1.23)  0.9 (0.6-1.4)  1.10 (1.00-1.22)  1.12 (0.86–1.44)  1.14 (0.98-1.32)  1.26 (0.93–1.68)  1.30 (1.00–1.66)  1.08 (1.04–1.12)  1.08 (1.06–1.10)  1.08 (1.06-1.10)  1.3 (1.1-1.5)  1.53 (1.40–1.66)  1.53 (1.47-1.58)  1.65 (1.43–1.90)  1.74 (1.49–2.04)  1.78 (1.59-2.00)  1.83 (1.65-2.03)  2 (1.8-2.7)  2.28 (1.83–2.85)  3.01 (2.87–3.15)  5.20 (5.11–5.30)  5.70 (5.57–5.83)  6.05 (6.00-6.11)  6.67 (6.32–7.03)  7.10 (6.71-7.50)  8.8 (8.7-9.0) | 0.74 (0.37-1.32)  1.06 (1.03-1.09)  1.08 (1.04-1.11)  1.08 (1.05-1.11)  1.20 (1.14–1.26)  2.16 (2.1-2.22) | 0.79 (0.51-1.15)  0.8 (0.5-1.3)  1.10 (0.96-1.27)  1.4 (1.1-1.6) | 0.7 (0.4-1.3)  1.9 (0.8-4.2) | 0.21 (0.01-1.18)  1.23 (0.98–1.53)  1.28 (1.14-1.43)  1.30 (1.18-1.43) | 1.1 (0.69–1.66)  1.15 (0.93-1.42)  1.17 (0.94-1.45) | 0.48 (0.34-0.67)  0.52 (0.38-0.69)  1 (0.62–1.53) | 0.79 (0.46–1.27)  0.94 (0.76-1.16)  1.05 (0.84-1.30)  1.9 (0.9-3.8)  2.2 (0.5-8.7) | 0.71 (0.56-0.88)  0.98 (0.93-1.04)  0.99 (0.94-1.04)  1.01 (0.96-1.06)  1.02 (0.85-1.22)  1.08 (0.98-1.18)  1.08 (0.98–1.2)  1.53 (1.36-1.72)  2.18 (2.07-2.31)  3.91 (3.41-4.45) |
| Other urological (e.g., bladder, kidney  TOTAL: 96  Lower: 0  Non-significant: 21  Higher: 75 | 1.0 (0.8-1.2)  1.04 (0.97–1.12)  1.16 (1.14-1.17)  1.22 (1.09–1.36)  1.22 (1.1-1.35)  1.24 (1.14-1.36)  1.29 (1.27–1.31)  1.30 (1.28-1.32)  1.31 (1.04-1.65)  1.32 (1.29-1.35)  1.40 (1.37–1.43)  1.54 (1.27–1.86)  1.55 (1.50–1.60)  1.56 (1.38-1.77)  1.70 (1.62–1.78)  2.0 (1.6-2.5)  2.20 (1.59-2.96)  2.39 (2.36-2.42)  3.2 (3.1-3.3)  44.5 (43.9–45.0) | 1.04 (1.00-1.07)  1.12 (0.97-1.29)  1.13 (1.09-1.16)  1.15 (1.13-1.17)  1.16 (1.13-1.19)  1.22 (1.20-1.24)  1.23 (1.14-1.32)  1.23 (1.19-1.27)  1.25 (1.21-1.28)  1.26 (1.2-1.33)  1.29 (1.18–1.4)  1.31 (1.29-1.33)  1.37 (1.34-1.41)  1.56 (1.49-1.64)  1.66 (1.42-1.93)  1.77 (1.38-2.23)  1.79 (1.64-1.94)  1.92 (1.72-2.14)  2.08 (1.89-2.29)  2.13 (2.09-2.16)  2.96 (2.47-3.52)  3.08 (2.99-3.17)  4.27 (3.84-4.75)  4.75 (1.54-11.09) | 1.0 (0.8-1.2)  2.0 (1.6-2.5) | 0.7 (0.3-1.6)  1.6 (0.8-3.3) | 1.14 (0.98-1.31)  1.33 (0.85–1.97)  1.05 (1.01-1.09)  1.21 (1.1-1.33)  1.29 (1.17-1.42)  1.63 (1.17-2.2)  1.71 (1.49-1.96)  1.86 (1.49-2.29)  1.89 (1.52-2.33)  2.15 (1.97-2.36)  2.25 (2.01-2.52)  2.37 (1.14-4.36)  2.96 (1.86-4.48) | 1.20 (0.85-1.65)  1.18 (1.09-1.28)  1.47 (1.34-1.61) | 0.82 (0.59-1.10)  1.31 (1.15-1.47)  1.65 (1.50-1.81) | 0.89 (0.56-1.35)  0.99 (0.71-1.34)  1.5 (0.5-4.8)  1.20 (1.02-1.38)  1.21 (1.02-1.41)  1.46 (1.14-1.85)  1.46 (1.28-1.66)  2.43 (1.44-3.84)  4.1 (1.7-9.9) | 0.91 (0.66-1.23)  0.95 (0.88-1.04)  0.96 (0.9-1.03)  0.96 (0.91-1)  1.02 (0.81–1.26)  1.02 (0.99-1.05)  1.77 (0.94-3.03)  1.11 (1.03-1.19)  1.12 (1.06-1.18)  1.13 (1.06-1.24)  1.18 (1.12-1.24)  1.20 (1.15-1.24)  1.21 (1.08-1.36)  1.23 (1.04-1.44)  1.23 (1.18-1.29)  1.28 (1.14-1.44)  1.71 (1.17-2.4)  2.05 (1.96-2.13)  2.51 (2.01-3.09)  2.56 (1.63-3.85) |
| Sarcomas  TOTAL: 17  Lower: 1  Non-significant: 9  Higher: 7 | 0.82 (0.73-0.91)  0.8 (0.3-2.4)  1.16 (0.95-1.40)  1.2 (0.9-1.5)  1.4 (0.9-2.2)  1.21 (1.15–1.27)  1.27 (1.06-1.51)  1.34 (1.06-1.67)  1.38 (1.21–1.59)  2.8 (1.8-4.4) | 1.38 (1.21–1.59) | 1.0 (0.7-1.3)  1.2 (0.7-2.1) | 1.4 (0.4-5.7)  2.8 (1.7-4.7) |  |  |  | 1.6 (0.4-6.6)  3.4 (0.5-24.4) |  |
| Head and neck cancer including salivary  TOTAL: 45  Lower: 1  Non-significant: 10  Higher: 34 | 0.94 (0.66-1.35)  1.1 (0.9-1.5)  1.13 (1.00-1.27)  1.2 (1.1–1.3)  1.4 (1.1-1.9)  1.4 (1.3–1.5)  1.42 (1.26-1.60)  1.5 (1.2-1.9)  1.8 (1.6–2.1)  1.80 (1.53-2.11)  1.81 (1.76–1.86)  1.90 (1.78–2.02)  2.09 (1.89–2.31)  2.4 (2.1-2.7) | 1.0 (0.9-1.1)  1.3 (1.0-1.8)  1.5 (1.2-1.9)  1.54 (1.49-1.59)  2.4 (2.1-2.8)  3.2 (3.1-3.29)  4.42 (4.26-4.58) | 1.2 (0.9-1.5)  1.3 (1.1–1.5)  1.3 (1.1–1.5)  1.3 (1.2–1.5)  1.4 (1.3–1.6)  1.5 (1.1–1.9)  1.6 (1.3–2.0) | 1.0 (0.4-2.7) | 3.80 (3.12-4.58) | 5.45 (3.62-7.87) | 5.21 (4.06-6.58) | 1.0 (0.1-7.1)  8.06 (6.15-10.37) | 0.8 (0.7-0.9)  1.1 (0.9–1.4)  1.6 (0.9-2.7)  1.4 (1.2–1.6)  1.62 (1.49-1.76)  1.62 (1.54-1.71)  2.1 (1.1-3.5)  2.2 (1.7–2.8)  2.3 (1.6-3.2)  3.02 (2.83-3.23)  4.28 (3.93-4.66) |
| Brain and other central nervous system (CNS)  TOTAL: 17  Lower: 0  Non-significant: 2  Higher: 15 | 1.0 (0.3-3.3)  1.4 (1.1-1.6)  2.14 (1.61-2.79)  2.21 (2.06–2.36)  2.60 (2.44-2.77)  3.32 (2.81-3.90)  3.62 (3.05–4.30)  5.00 (4.73-5.28) | 1.88 (1.32–3.26)  1.94 (1.78–2.10) | 1.3 (1.1-1.6) | 1.4 (0.9-2.4) |  |  |  | 2.4 (1.1-5.3) | 3.60 (3.14–4.11)  5.66 (5.15-6.21)  6.55 (4.15–9.83)  9.63 (8.19-11.25) |
| Skin and melanoma  TOTAL: 44  Lower: 11  Non-significant: 15  Higher: 18 | 0.5 (0.4-0.6)  0.52 (0.44-0.60)  0.54 (0.47-0.63)  0.82 (0.66-1.02)  0.94 (0.89-1.00)  0.98 (0.96-1.00)  1.10 (1.03–1.18)  1.29 (1.14-1.45)  1.33 (1.28–1.38) 1.44 (1.33-1.57)  1.98 (1.93-2.03)  8.18 (8.16-9.48) | 0.88 (0.86-0.90)  0.91 (0.89–0.93)  1.49 (1.4–1.59)  2.04 (1.99-2.1)  2.28 (1.99–2.61) | 0.4 (0.3-0.6)  0.93 (0.91-0.95)  1.15 (1.10-1.21) | 0.8 (0.4-1.4) | 1.03 (0.93–1.13)  1.26 (0.87–1.76)  3.52 (1.92–5.9) | 0.99 (0.03–5.54)  1.12 (0.93–1.33)  2.32 (1.35–3.72) | 0.75 (0.63–0.89)  1.03 (0.51–1.85)  2.52 (0.69–6.46) | 0.78 (0.64–0.94)  0.94 (0.90-0.99)  0.9 (0.3-2.9)  1.21 (0.61–2.17)  1.15 (1.04-1.27)  3.76 (1.22–8.78) | 0.93 (0.90-0.97)  0.98 (0.94-1.02)  0.99 (0.95–1.04)  1 (0.96-1.04)  1.10 (1.02-1.19)  1.67 (1.46–1.91)  1.95 (1.84-2.06)  3.34 (2.57–4.26) |
| Thyroid  TOTAL: 30  Lower: 11  Non-significant: 7  Higher: 12 | 0.55 (0.40-0.75)  0.57 (0.55–0.59)  0.75 (0.64-0.87)  0.84 (0.73-0.96)  0.87 (0.84–0.91)  0.91 (0.72–1.15)  1.0 (0.7-1.3)  1.14 (1.06–1.23)  1.21 (1.04–1.41)  1.47 (1.30–1.67)  1.59 (1.55-1.64)  5.66 (4.31–7.43) | 0.57 (0.45-0.69)  0.63 (0.54-0.71)  0.77 (0.73-0.82)  0.31 (0.04-1.12)  1.54 (1.49-1.59) | 0.34 (0.11-0.80)  0.8 (0.6-1.2) | 1.1 (0.5-2.6) | 0.29 (0.04-1.06)  2.41 (2.06-2.83) | 1.97 (1.54-2.52) | 1.52 (1.21-1.90) | 1.24 (0.92-1.68)  3.5 (1.3-9.3) | 0.61 (0.40-0.88)  0.79 (0.73-0.86)  1.76 (1.64-1.88)  2.66 (2.36-2.98) |
| Other cancers  (neuroblastoma, retinoblastoma, neuroendocrine tumours)  TOTAL: 23  Lower: 1  Non-significant: 9  Higher: 13 | 1.00 (0.65-1.54)  1.3 (0.9–1.3)  1.20 (1.14-1.26)  1.36 (1.24–1.50)  1.5 (1.1-2.1)  1.58 (1.34-1.86)  1.65 (1.54-1.77)  1.66 (1.55–1.78)  2.36 (2.17-2.57)  4.95 (1.35-12.67) | 1.18 (1.12-1.25)  2.31 (2.20-2.42) | 1.4 (1.0-2.0) | 1.9 (0.8-4.6) | 1.42 (1.10-1.81) | 0.74 (0.34-1.41) | 1.08 (0.66-1.66) | 0.9 (0.2-3.7)  3.0 (0.8-12.0)  1.74 (1.15-2.54) | 0.83 (0.77-0.9)  1.1 (0.7–1.5)  1.21 (1.07-1.36) |

Green font represents lower risk of CVD death (SMR<1), black font represents an equivalent risk (95%CI of SMR included 1), red font represents higher risk of CVD death (SMR >1).
