# Supplementary material for: Cardiovascular mortality in people with cancer compared to the general population: A systematic review and meta‐analysis
Source: Cancer Med. 2024 Aug 3;13(15):e70057. doi: 10.1002/cam4.70057 (PMC11297437; doi:10.1002/cam4.70057)
Supplement: Supplementary file 4 — Table S3. [file CAM4-13-e70057-s002.docx]

**Supplementary Material** Assessment of study quality and risk of bias summary

|  | **Score** | | | | |
| --- | --- | --- | --- | --- | --- |
| **Study** | **Selection** | **Comparability** | **Outcome** | **Total** |  |
| Dores 2020, Gad 2019, Yang 2023a, Yin 2022b | 🟆🟆🟆🟆 | 🟆🟆 | 🟆🟆🟆 | 9 |  |
| Afifi 2021, Afifi 2020, Afify 2023, Anderson 2021, Anderson 2019, Appiah 2021, Arora 2019, Awad 2022, Baade 2006, Beadle 2013, Beard 2013, Caocci 2020, Chen 2023, Chen 2022a, Chen 2022b, Chen 2022c, Chen 2021, Chen 2020, Chi 2023, Dai 2022, Dalal 2020, de Vries 2021, Du 2021, Eisfeld 2023, Elgenidy 2023, Elgenidy 2022, Elmehrath 2021a, Elmehrath 2021b, Eriksson 2000, Fang 2010, Felix 2017, Forster 2022, Fossa 2004, Fossa 2007, Fung 2015, Gaitanidis 2019, Gon 2023, Guan 2023, Guan 2022, Guan 2021, Guo 2022, Guo 2020, Harashima 2021, Hellesnes 2021, Henson 2016, Hisada 2007, Jensen 2008, Jin 2021, Jung 2023, Kim 2022, Koczwara 2021, Kurisu 2022, Kvammen 2019, Li 2022, Liu 2022, Lou 2023, Low 2019, Lu 2022, Lu 2021, Mangone 2021, Massa 2017, Miao 2022, Moke 2021, Oh 2020, Peng 2022, Shi 2021, Shin 2010, Sonbol 2023, Song 2022a, Stoltzfus 2020, Sturgeon 2019, Suh 2020, Su 2022, Sun 2021, Swerdlow 2007, Udkoff 2023, van Hemelrijck 2010, van Monsjou 2016, Vo 2023, Vo 2022, Wang 2023, Wang 2022a, Wang 2022b, Wang 2022c, Weberpals 2018, Weiner 2021, Wild 2007, Wu 2022, Xia 2022, Xie 2022, Xue 2023, Yang 2023b, Yang 2021a, Yang 2021b, Yao 2023, Ye 2022, Ye 2019, Yin 2023, Yin 2022, Yin 2019, Yu 2022, Yu 2012, Zang 2020, Zaorsky 2019, Zaorsky 2017, Zar 2008, Zhai 2020, Zheng 2022 | 🟆🟆🟆 | 🟆🟆 | 🟆🟆🟆 | 8 |  |
| Abuamsha 2019, Agha 2022, Ameijide 2019, Cheng 2022, Feng 2021, Katuwal 2021, Levi 2002, Li 2019, Liu 2023, Lyu 2022, Sadeq 2023, Song 2022b, Wang 2021a, Wang 2021b, Zhang 2021 | 🟆🟆🟆 | 🟆 | 🟆🟆🟆 | 7 |  |
| Eifler 2012, Hooning 2006, Otto 2006, Zagars 2004 | 🟆🟆 | 🟆🟆 | 🟆🟆🟆 | 7 |  |
| Ascoli 2009, Harvitkar 2021, Howlader 2017, Youn 2014 | 🟆🟆🟆 |  | 🟆🟆🟆 | 6 |  |
| Lee 2000 | 🟆🟆 | 🟆🟆 | 🟆🟆 | 6 |  |

**Note: A study can be awarded a maximum of one score for each numbered item within the Selection and Outcome categories. A maximum of two score can be given for Comparability**

**Selection (Representativeness of the exposed cohort)**

(a) truly representative of the average cancer patient in the community *

(b) somewhat representative of the average cancer patient in the community *

(c) selected group of users, e.g. nurses, volunteers

(d) no description of the derivation of cohort

**Selection (Selection of the non-exposed cohort)**

(a) drawn from the same community as the exposed cohort *

(b) drawn from a different source

(c) no description of the derivation of the non exposed cohort

**Selection (Ascertainment of exposure: cancer status)**

(a) secure record (eg surgical records) *

(b) structured interview *

(c) written self report

(d) no description

**Selection (Demonstration that CVD death was not present at the start of the study)**

(a) yes *

(b) no

**Comparability (on the basis of the design or analysis)**

(a) study controls for age *

(b) study controls for sex *

**Outcome (Assessment of outcome)**

(a) independent blind assessment *

(b) record linkage *

(c) self report

(d) no description

**Outcome (Was follow-up long enough for outcomes to occur)**

(a) yes (select an adequate follow up period for outcome of interest) *

(b) no

**Outcome (Adequacy of follow-up of cohorts)**

(a) complete follow up - all subjects accounted for *

(b) subjects lost to follow up unlikely to introduce bias - small number lost - >80 % (select an adequate %) follow up, or description provided of those lost) *

(c) follow up rate < 80% (select an adequate %) and no description of those lost

(d) no statement
