# Supplementary material for: Cardiovascular mortality in people with cancer compared to the general population: A systematic review and meta‐analysis
Source: Cancer Med. 2024 Aug 3;13(15):e70057. doi: 10.1002/cam4.70057 (PMC11297437; doi:10.1002/cam4.70057)
Supplement: Supplementary file 5 — File S1. [file CAM4-13-e70057-s003.docx]

**Supplementary material**

Full search term for OVID Medine

Includes Medline (1946- ) and the database segments Epub Ahead of Print, In-Process, In-Data-Review, and Other Non-Indexed Citations

| **#** | **Searches** |
| --- | --- |
| 1 | exp Neoplasms/ or cancer survivors/ |
| 2 | (cancer* or neoplas* or malignan* or tumor* or tumour* or sarcoma* or leuk?emia* or lymphoma*).tw,kw. |
| 3 | 1 or 2 |
| 4 | Mortality/ |
| 5 | mortality.fs. |
| 6 | (mortality or death or decedent*).tw,kw. |
| 7 | or/4-6 |
| 8 | ((standard* adj mortality) or SMR?).tw,kw. |
| 9 | 3 and 7 and 8 |
| 10 | limit 9 to (English language and yr="2000 -Current") |
| 11 | (Comment or congress or conference or editorial or letter or news).pt. |
| 12 | 10 not 11 |

Notes:

- / = Medical Subject Heading (MeSH) term search
- fs = MeSH term subheading search
- tw = search on title and abstract
- kw = search on author keywords field
- pt = publication type search
- exp = exploded MeSH term search (main heading term plus narrower concept MeSH terms)
- * = truncation (retrieves variations on a word stem)
- ? = allows substitution of 0 or 1 character
- adj = retrieves citations where search terms occur next to each other
